# Internal Resonance Mismatch‐Controlled Dissipative Bistability Collapse in Coupled Micromechanical Oscillators

**DOI:** 10.1002/advs.77439

**Published:** 2026-08-30

**Authors:** Jianlin Chen, Xin Zhou, Takashiro Tsukamoto, Nan Wang, Shuji Tanaka

**Affiliations:** ^1^ School of Microelectronics Shanghai University Shanghai China; ^2^ School of Microelectronics Southern University of Science and Technology (SUSTech) Shenzhen China; ^3^ Department of Robotics Tohoku University Sendai Miyagi Japan

**Keywords:** Bistability, Dissipative system, Duffing equation, Internal resonance, MEMS sensors, Multimode interference, Nonlinear resonance

## Abstract

Nonlinear resonance governs the stability boundaries of high‐Q micromechanical systems, but the collapse of Duffing bistability is usually limited by fixed intrinsic dissipation. Here we demonstrate internal resonance mismatch‐controlled dissipative bistability collapse (DBC) in serially coupled micromechanical oscillators. By independently tuning an antiphase internal mode near a 1:2 internal resonance with a driven rotational mode, we activate a nonlinear energy‐transfer pathway without directly changing the drive strength. Frequency responses and frequency–mismatch maps show that the high‐amplitude Duffing branch collapses within a finite mismatch window around the near‐zero internal‐resonance condition, accompanied by a sharp downshift of the jump frequency and an approximately 80% reduction in stored vibration energy. Phase‐resolved measurements reveal that the internal‐mode resonance separates two phase domains, indicating a mismatch‐controlled change in how internal‐mode feedback is projected onto the driven‐mode dynamics. Near the DBC transition, strong internal‐mode excitation coincides with a favorable dissipative phase projection, maximizing phase‐weighted internal‐resonance feedback. Effective damping extraction and energy‐balance modeling further show that the collapse is caused by the rapid growth of the internal resonance mediated dissipative contribution, rather than by intrinsic damping alone. These results establish internal resonance mismatch as an independent control axis for nonlinear dissipation engineering and reconfigurable micromechanical switching.

## Graphical abstract

This paper reveals internal resonance mismatch‐controlled dissipative bistability collapse in a coupled micromechanical resonator. Tuning *δ*_IR_ changes both internal‐mode amplitude and phase‐dependent feedback. For negative mismatch, Duffing bistability survives. Near *δ*_IR_ ≈ 0, strong internal‐mode excitation and favorable dissipative phase projection maximize IR‐induced damping, collapsing the high‐amplitude branch. Positive mismatch yields suppressed but sustained bistability.

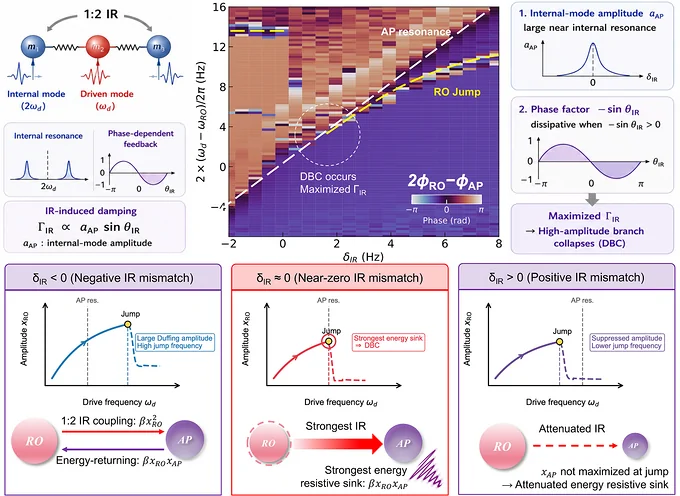

## 1 Introduction

Nonlinear dynamics in high‐quality‐factor resonators underpin a wide range of physical systems, acting as a bridge between classical and quantum phenomena. These dynamics are fundamental to fields ranging from cavity optomechanics [1, 2, 3, 4] and superconducting circuits [5, 6] to micro‐ and nano‐electromechanical systems (MEMS/NEMS) [7, 8]. Among the various nonlinear manifestations, Duffing nonlinearity represents a canonical paradigm, where intrinsic geometric or material nonlinearities give rise to amplitude‐dependent resonance shifts, hysteresis, and bistability [9]. Historically viewed as a limitation to dynamic range, such nonlinear responses have recently been harnessed as functional resources for advanced applications, including bifurcation‐based sensing [10, 11, 12], mode‐localization sensing [13, 14], signal processing and amplification [15, 16, 17], mechanical logic and memory [18, 19, 20], frequency‐comb generation [21, 22, 23], and reservoir computing [24]. In the quantum regime, nonlinear nanomechanical resonators also provide routes toward ground‐state cooling and quantum control [25, 26, 27, 28]. Across these contexts, the practical utility of nonlinear dynamics depends critically on the ability to actively shape and reconfigure the stability landscape.

In conventional Duffing resonators, this capability remains fundamentally constrained. The width and robustness of the bistable regime are largely determined by intrinsic dissipation and nonlinear loss mechanisms, which are fixed once the device is fabricated [9]. Although the onset of bistability can be shifted by changing drive amplitude, drive‐frequency detuning, or feedback control [29, 30], the collapse of the high‐amplitude state remains governed by the available dissipation channels. This rigidity limits real‐time and deterministic control of nonlinear stability boundaries, which is essential for adaptive signal processing, mechanical logic, and reconfigurable filtering [31].

Internal resonance (IR) provides a promising route to overcome this limitation by enabling energy exchange between a driven mode and an internal mode under near‐commensurate frequency conditions, such as 1:2 or 1:3 ratios [32, 33, 34, 35]. In coupled oscillators, such modal interactions have been shown to generate rich nonlinear dynamics, including frequency stabilization [29], synchronization [36, 37], and coherent energy transfer [38, 39, 40, 41]. In particular, theoretical studies predict that coupling to a higher‐frequency internal mode can appear as IR‐enhanced nonlinear damping for the driven mode [9, 42]. Experimental signatures of such damping have been reported in carbon nanotube resonators [43, 44] and inferred from transient ringdown dynamics in graphene drums [45]. More recently, parametric pumping has been used to tune nonlinear damping in graphene nanoresonators [46]. These studies established internal‐resonance‐induced damping as an important nonlinear dissipation mechanism.

However, the feedback pathway responsible for IR‐induced damping remains insufficiently resolved. In many previous systems, the internal mode is not independently accessible during steady‐state operation, and damping enhancement is inferred mainly from amplitude saturation, linewidth broadening, or transient decay [43, 46]. As a result, IR‐induced damping is often treated as an effective phenomenological loss term. The missing information is the internal phase. The amplitude of the internal mode identifies where energy transfer occurs, but it does not determine how the internal‐mode response is projected back onto the driven‐mode dynamics [47]. Depending on the relative phase, the feedback can act mainly as a reactive perturbation of the Duffing backbone or as a dissipative projection that enhances the effective damping [48, 49, 50, 51]. Thus, phase‐resolved access to both modes is essential for converting internal resonance‐induced damping from a phenomenological observation into a controllable feedback mechanism.

Here, we demonstrate an IR‐mismatch‐controlled route to dissipative bistability collapse (DBC) in coupled micromechanical oscillators. The controlled parameter is the internal‐resonance mismatch, *δ*_IR_ = (*ω*_AP_ */*2 − *ω*_RO_)*/*2*π*, which quantifies the frequency offset between half of the anti‐phase‐mode (AP) frequency and the rotational‐mode (RO) frequency. Thus, *δ*_IR_ ≈ 0 corresponds to the 1:2 internal‐resonance condition, *ω*_AP_ ≈ 2*ω*_RO_. Using a serially coupled triple‐resonator platform [52, 53, 54], we electrostatically tune this mismatch while preserving simultaneous access to the RO mode and the AP internal mode. This architecture allows us to shift the IR condition relative to the Duffing response and to map the nonlinear stability landscape across negative, near‐zero, and positive mismatch regimes.

A central advance of this work is the phase‐resolved identification of the AP feedback mechanism. By simultaneously measuring the RO mode response at *ω_d_* and the AP response at 2*ω_d_*, we reconstruct the internal phase mismatch, *θ*_IR_ = wrap[2*ϕ*_RO_(*ω_d_*) − *ϕ*_AP_(2*ω_d_*)]. The resulting phase map reveals a clear topology: the AP resonance approximately separates two internal‐phase domains, indicating that the feedback projection changes when the system crosses the internal‐mode resonance. Near zero IR mismatch, strong AP excitation and favorable dissipative phase projection occur where the Duffing branch is most sensitive to additional damping, producing a sharp increase in the IR‐mediated AP‐channel dissipative contribution. This enhanced dissipation suppresses the high‐amplitude branch, downshifts the jump frequency, and collapses the stored vibration energy. By linking phase topology, amplitude collapse, and damping decomposition, this work establishes IR mismatch as an independent control axis for dissipation engineering in coupled nonlinear oscillators [55, 56, 57].

## 2 Results

### 2.1 Device Architecture and Tunable Internal Resonance

**FIGURE 1:**
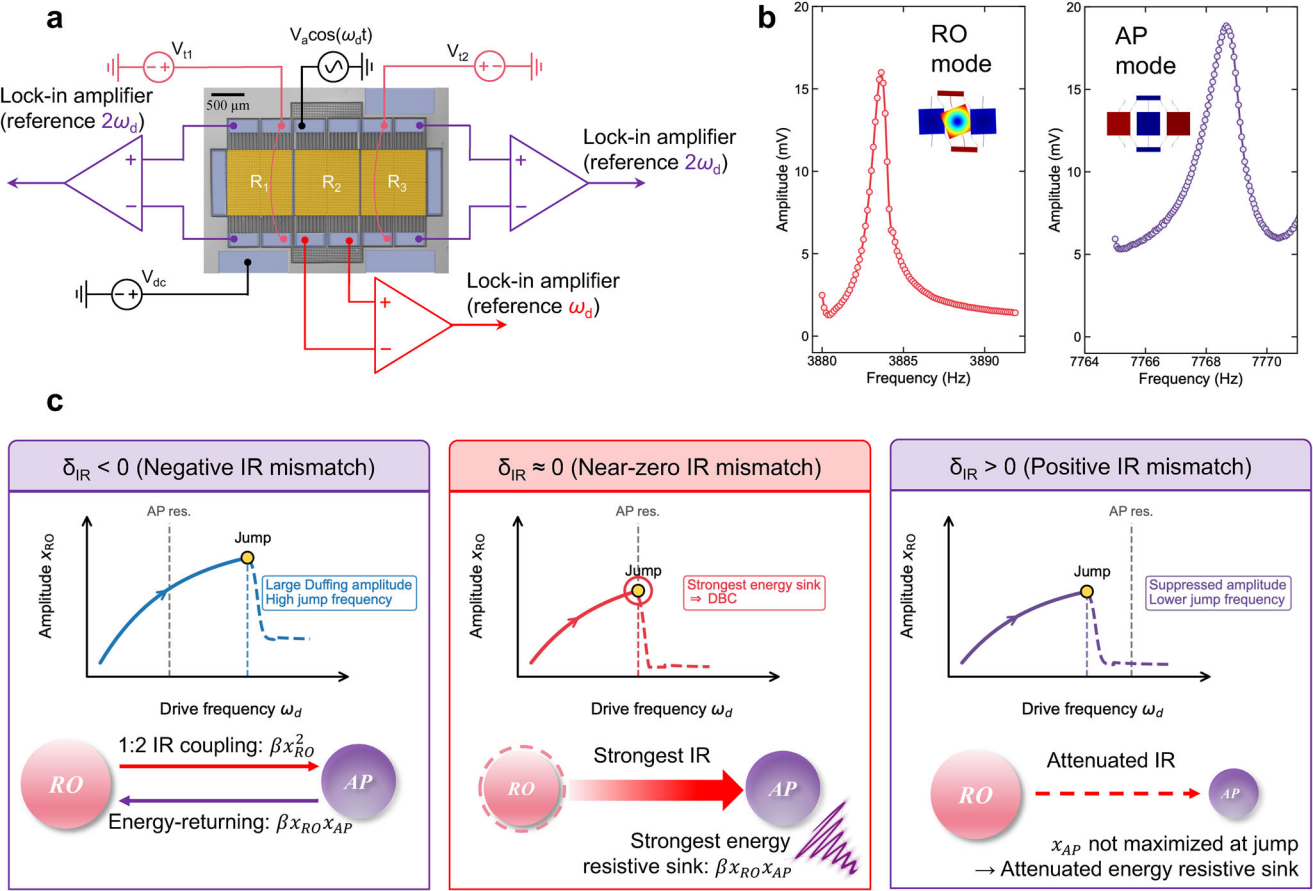
Experimental platform and mechanism of IR‐mismatch‐controlled dissipative bistability collapse. (a) False‐color scanning electron microscope (SEM) micrograph and electrical measurement schematic of the serially coupled triple‐resonator device. The structure consists of three mechanically coupled resonators, *R*_1_, *R*_2_, and *R*_3_. The central resonator is electrostatically driven at the fundamental frequency *ω_d_* and detected by a lock‐in amplifier referenced to *ω_d_*, while the two outer resonators are differentially sensed by lock‐in amplifiers referenced to 2*ω_d_*. This configuration enables simultaneous measurement of the rotational (RO) response and the anti‐phase (AP) internal‐mode response. (b) Measured linear frequency responses and finite‐element mode shapes of the two interacting modes involved in the 1:2 internal resonance. The RO mode is mainly localized in the central resonator, whereas the AP mode corresponds to out‐of‐phase motion of the two outer resonators. Their near‐commensurate frequency relation establishes the internal‐resonance pathway through which energy can be transferred from the driven RO mode to the AP mode. (c), Schematic illustration of the dissipative bistability collapse (DBC) mechanism controlled by the internal‐resonance mismatch *δ*_IR_. Under negative IR mismatch (*δ*_IR_ *<* 0), the AP feedback near the RO Duffing jump is energy‐returning, allowing the large‐amplitude Duffing branch and high jump frequency to survive. Under near‐zero IR mismatch (*δ*_IR_ ≈ 0), strong AP excitation coincides with a dissipative feedback condition near the RO resonance. The AP mode therefore acts as the strongest energy resistive sink, producing large IR‐enhanced damping and collapsing the high‐amplitude bistable branch. Under positive IR mismatch (*δ*_IR_ *>* 0), the AP feedback *x*_AP_ remains dissipative but is attenuated, leading to reduced RO amplitude and a lower jump frequency.

A serially coupled triple‐resonator device, shown in Figure 1a, is employed to achieve frequency decoupling between two modes involved in a 1:2 IR. The RO mode, shown in Figure 1b, is actuated by an AC voltage applied to the central resonator, so that the vibration energy is mainly concentrated in the central mass. In contrast, the AP mode, also shown in Figure 1b, consists of out‐of‐phase motion of the two outer resonators, while the central resonator remains nearly stationary. The device was measured in a vacuum chamber at approximately 25 Pa. In the linear regime, the quality factors of the RO and AP modes were both approximately 3000, mainly limited by air damping.

During the measurement, a DC bias of 10 V was applied to the proof mass, and an AC driving signal *V_a_* cos(*ω_d_t*) was applied to the parallel‐plate electrodes of the central resonator *R*_2_. Two additional DC tuning biases were applied to *R*_1_ and *R*_3_, respectively, to compensate for stiffness mismatch and to electrostatically tune the AP‐mode frequency. Since the RO‐mode motion is mainly localized in *R*_2_, the RO response was obtained from the differential parallel‐plate sensing electrodes of *R*_2_ and demodulated at the drive frequency *ω_d_*. In contrast, the AP‐mode motion is concentrated in the two outer resonators. Therefore, under balanced anti‐phase motion, the AP response was reconstructed from the averaged outer‐resonator signals measured from *R*_1_ and *R*_3_ and demodulated at 2*ω_d_*. Frequency‐sweep measurements were performed using a Zurich Instruments UHFLI lock‐in amplifier.

The measured frequency responses confirm that the two modes have a near‐commensurate frequency relation, *ω*_AP_ ≈ 2*ω*_RO_, which provides the condition for 1:2 IR. Owing to the tripleresonator architecture, the AP‐mode frequency can be tuned independently of the RO mode through electrostatic softening of the outer suspension beams. As the DC tuning bias applied to the outer resonators increases, the AP‐mode frequency decreases gradually, while the RO‐mode frequency remains nearly unchanged. Specifically, the RO mode exhibits a frequency shift of only approximately 100 ppm across the applied bias range, which is negligible compared with the substantial tuning range of the AP mode (changed by 3000 ppm). As a result, *ω*_AP_ can be continuously tuned until the exact 1:2 IR condition, *ω*_AP_ = 2*ω*_RO_, is reached.

We parameterize the system using the IR mismatch, *δ*_IR_ = (*ω*_AP_ */*2 − *ω*_RO_)*/*2*π*, where *ω*_RO_ and *ω*_AP_ are the linear resonance frequencies of the RO and AP modes, respectively. By sweeping *δ*_IR_, we tune the relative position of the AP internal resonance with respect to the RO resonance, thereby controlling the strength and phase of the IR feedback without significantly changing the RO‐mode resonance or other nonlinear coefficients.

Figure 1c summarizes the physical mechanism of IR‐mismatch‐controlled dissipative bistability collapse (Note S2, Equations S33–S36). For *δ*_IR_ *<* 0, the AP response is activated before the RO resonance, and then the AP feedback to RO Duffing response is energy‐returning, so the large‐amplitude Duffing branch remains stable, and the jump occurs at a relatively high frequency. For *δ*_IR_ ≈ 0, the AP internal resonance is aligned with the RO resonance, which causes the RO→AP energy transfer through the 1:2 IR coupling to become strongest, and the AP mode acts as a resistive energy sink. This produces the largest IR‐enhanced damping, rapidly dissipates energy from the RO mode, and collapses the high‐amplitude bistable branch, which we refer to as DBC. For *δ*_IR_ *>* 0, the AP response is shifted to the high‐frequency side of the RO resonance. The AP feedback *x*_AP_ remains dissipative but becomes attenuated, resulting in a suppressed RO amplitude and a lower jump frequency rather than the strongest collapse. Therefore, DBC occurs when strong AP internal resonance and dissipative feedback coincide near the RO resonance under near‐zero IR mismatch.

### 2.2 Observation of Dissipative Bistability Collapse

**FIGURE 2:**
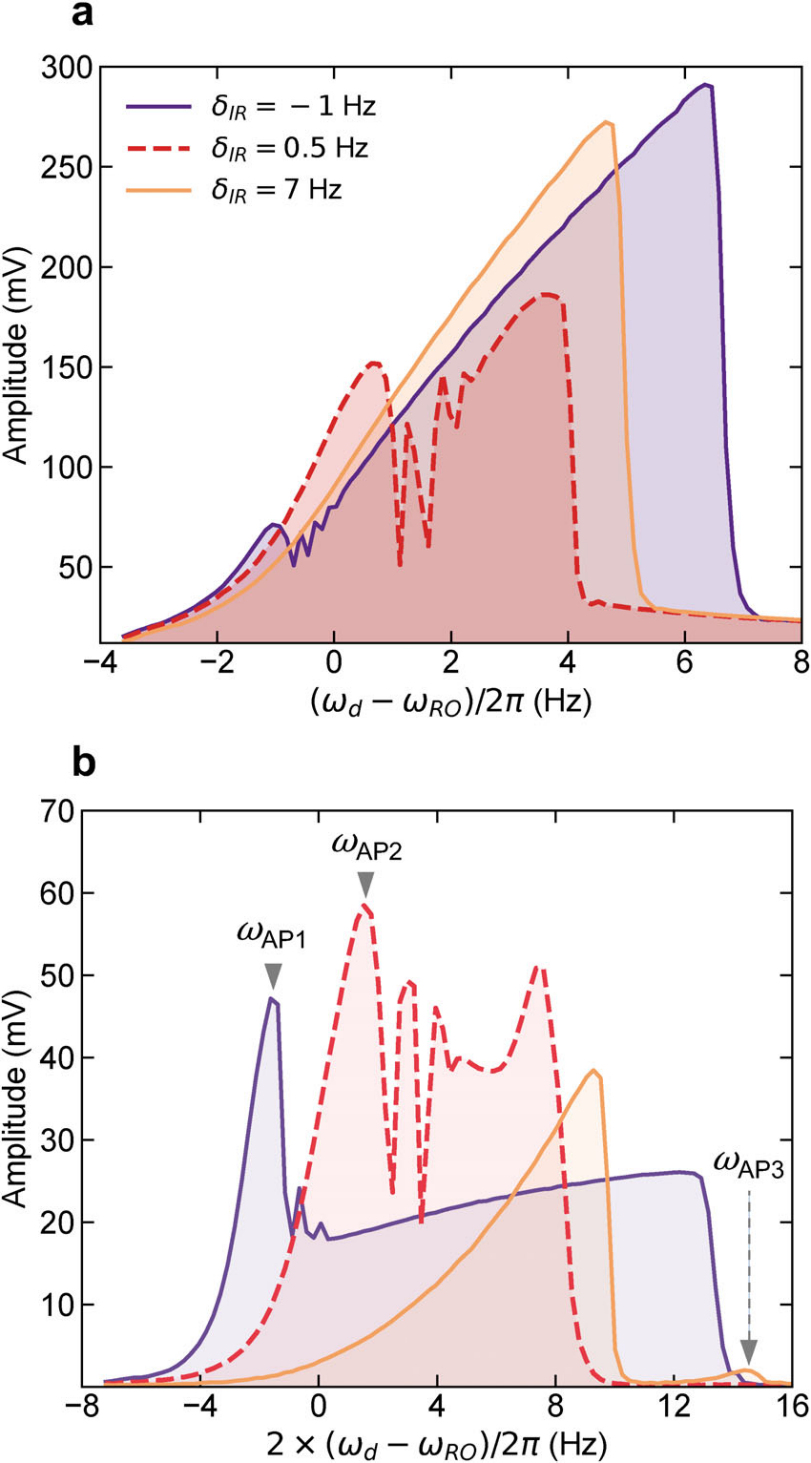
Frequency response evolution near the 1:2 IR. (a) Measured nonlinear frequency response of the RO mode, *x*_RO_(*ω_d_*), under different IR mismatch conditions. For *δ*_IR_ = −1 Hz, the response is dominated by a conventional Duffing‐type high‐amplitude branch with a pronounced jump at high drive frequency. As the IR mismatch approaches the near‐zero regime, *δ*_IR_ = 0.5 Hz, the high‐amplitude branch is strongly destabilized and breaks into a collapsed response, indicating DBC. For *δ*_IR_ = 7 Hz, the RO response remains continuous over a broader frequency range but exhibits IR‐induced damping and modified jump behavior. (b) Simultaneously measured AP response, *x*_AP_(2*ω_d_*), for the same IR mismatch conditions. The AP response reveals how the internal mode is activated by the second‐harmonic component of the RO motion. The arrows mark the frequency positions of the AP resonances, *ω*_AP1_, *ω*_AP2_, and *ω*_AP3_, corresponding to the three IR mismatch settings.

To realize IR coupling between the RO and AP modes, the RO mode is driven into the nonlinear regime while the modal frequency ratio is tuned close to 1:2 (see Note S1 and FigureS1). Figure 2 shows the evolution of the RO response *x*_RO_(*ω_d_*) and the AP response *x*_AP_(2*ω_d_*) as the IR mismatch *δ*_IR_ is swept across the 1:2 IR condition. Here, *x*_RO_(*ω_d_*) is defined as the output of *R*_2_(*ω_d_*) because the RO vibration is localized mainly in the central resonator *R*_2_. Similarly, *x*_AP_(2*ω_d_*) is reconstructed from the outer‐resonator response at 2*ω_d_*. Under balanced AP motion, *R*_1_(2*ω_d_*) and *R*_3_(2*ω_d_*) are symmetrically tuned, while the central resonator has negligible superharmonic response, *R*_2_(2*ω_d_*) ≈ 0, as confirmed in Figure S2.

For negative IR mismatch, *δ*_IR_ *<* 0, the system remains in a Duffing‐dominant regime, where inter‐modal energy exchange is relatively weak (see Note S4 and Figure S1). The RO mode exhibits a standard hardening Duffing response: as the drive frequency *ω_d_* increases, *x*_RO_ follows the high‐amplitude backbone and eventually terminates in a sharp discontinuous drop at the jump frequency. Along this high‐amplitude branch, the AP response shows a Lorentzian‐like peak when *ω_d_* ≈ *ω*_AP_ */*2, corresponding to weak resonant energy transfer from the RO mode to the AP mode. This energy transfer produces only a small dip in the RO response, and the Duffing bistability remains largely intact.

When the IR mismatch approaches the near‐zero regime, *δ*_IR_ ≈ 0, the system enters the DBC regime. In this case, the large and stable high‐amplitude RO branch is strongly suppressed, and the bistable region abruptly collapses. As a result, the jump frequency shifts sharply to a lower value. Meanwhile, the AP response is strongly enhanced and reaches its maximum amplitude, indicating that energy transfer into the AP mode becomes most efficient. The AP response also develops a more complex structure, including Hopf‐type amplitude and phase modulation, as discussed in Note S4.2. These observations indicate that near‐zero IR mismatch converts the AP mode into a strong dissipative channel that destabilizes the RO high‐amplitude branch.

For positive IR mismatch, *δ*_IR_ *>* 0, the system enters an IR‐damped regime and does not recover the Duffing‐dominant response observed for *δ*_IR_ *<* 0. The RO response still shows a hardening Duffing‐like trend, but its amplitude is suppressed, and the jump frequency remains clamped at a value significantly lower than that of the negative‐mismatch case. The AP response changes from the sharp high‐amplitude peak observed near *δ*_IR_ ≈ 0 to a broader and lower‐amplitude plateau. Its amplitude drops concurrently with the RO jump, indicating that the AP‐mediated damping is weakened after the collapse. A small AP resonant peak can still be observed near *ω_d_* ≈ *ω*_AP_ */*2 beyond the RO jump frequency, as discussed in Note S4.3.

**FIGURE 3:**
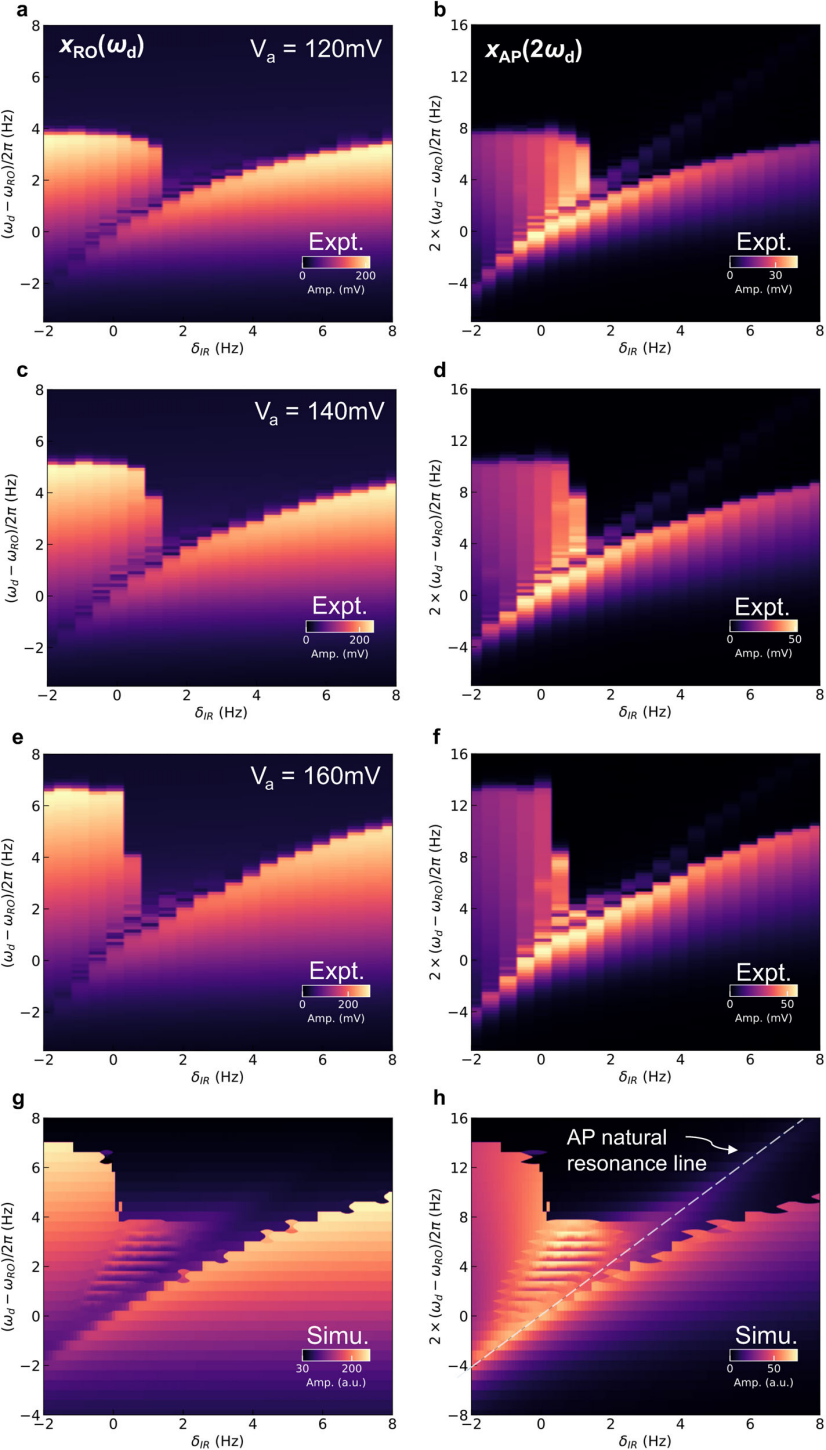
Experimental and numerical maps of the nonlinear RO and AP responses across IR mismatch. (a–f) Experimental nonlinear frequency–mismatch response maps of the RO response *x*_RO_(*ω_d_*) and AP response *x*_AP_(2*ω_d_*) as the IR mismatch *δ*_IR_ is swept from negative to positive values at three drive voltages, *V_a_* = 120, 140, and 160 mV. Panels (a, c, e) show the RO response, while panels (b, d, f) show the corresponding AP response. In the RO maps, the bright ridge marks the high‐amplitude Duffing branch and its jump boundary. For negative IR mismatch, this ridge remains nearly flat, indicating a Duffing‐dominant response with weak IR‐mediated energy transfer. As *δ*_IR_ approaches the near‐zero regime, the ridge collapses to lower frequency and develops alternating bright–dark bands, indicating DBC accompanied by Hopf‐type envelope modulation. For positive IR mismatch, the RO response is suppressed, and the jump frequency remains shifted to lower values, corresponding to an IR‐damped regime. In the AP maps, the IR‐enhanced response peak follows the AP resonance condition for negative mismatch and then becomes locked to the RO jump region as the mismatch approaches and crosses the near‐zero regime. (g, h) Numerical nonlinear frequency–mismatch response maps of *x*_RO_(*ω_d_*) and *x*_AP_(2*ω_d_*) obtained by solving Equation (1) using a classical fourth‐order Runge–Kutta method. The simulations reproduce the main experimental features, including the collapse of the RO Duffing branch, the enhanced AP response near the transition, and the oscillatory horizontal band structures in the transition region.

By mapping the frequency‐response curves against the IR mismatch *δ*_IR_, the DBC transition becomes directly visible as an abrupt reshaping of the nonlinear stability landscape, as shown in Figure 3. For a fixed drive voltage *V_a_*, the bright ridge in the RO colormap marks the high‐amplitude Duffing branch and its terminal jump frequency. For negative IR mismatch, *δ*_IR_ *<* 0, the RO response remains Duffing‐dominant: the maximum amplitude and jump frequency are nearly unchanged, consistent with a conventional hardening Duffing oscillator. A diagonal dark trace intersects the hardening backbone, corresponding to the AP resonance condition *ω_d_* ≈ *ω*_AP_ */*2. This feature indicates weak IR‐mediated energy transfer from the RO mode to the AP mode, but the energy transfer is not strong enough to destroy the Duffing bistability.

As the system approaches near‐zero IR mismatch, *δ*_IR_ ≈ 0, the RO response enters the DBC regime. The jump frequency and maximum amplitude drop abruptly, indicating collapse of the high‐amplitude Duffing branch and a sudden reduction of stored RO energy. Alternating bright–dark bands appear near the transition, revealing strong amplitude modulation associated with Hopf‐type dynamics. For positive IR mismatch, *δ*_IR_ *>* 0, the bright ridge gradually shifts upward again, showing partial recovery of the Duffing branch. However, the jump frequency remains strongly suppressed compared with the negative‐mismatch regime, indicating that IR‐mediated damping continues to constrain the RO stability boundary.

The AP colormaps in Figure 3b,d,f show the corresponding activation of the AP mode. For *δ*_IR_ *<* 0, the main bright line follows the AP resonance near *ω_d_* ≈ *ω*_AP_ */*2. As *δ*_IR_ approaches zero, the AP response becomes strongly enhanced, confirming that the RO–AP energy exchange is maximized near the DBC transition. For *δ*_IR_ *>* 0, the dominant AP feature no longer simply follows the AP natural resonance; instead, it becomes linked to the RO jump boundary because the AP mode is driven by the nonlinear RO motion. When the RO mode jumps down, the AP amplitude drops simultaneously. A weaker secondary trace at higher frequency continues to indicate the AP resonance, while the main AP response gradually weakens as the IR mismatch increases.

Increasing the drive voltage from 120 to 140 and 160 mV reveals the competition between Duffing hardening and IR‐mediated damping. For *δ*_IR_ *<* 0, the larger drive voltage increases the RO amplitude and shifts the Duffing jump frequency upward. However, the DBC transition remains pinned near *δ*_IR_ ≈ 0. Consequently, the contrast between the high‐energy Duffing state and the collapsed DBC state becomes more pronounced at higher drive voltages, producing a larger drop in the jump frequency at the transition.

The system dynamics are captured by the coupled nonlinear equations (see details in Note S2, Equations S11 and S12)

(1) $$\begin{array}{ccc} & & \ddot{\mu }+\omega_{1}^{2}u+2\varepsilon \mu_{1}\dot{u}+2\varepsilon \mu_{\mathrm{non}}\dot{u}u^{2}+2\varepsilon \beta \mathrm{uv}+3\varepsilon \gamma uv^{2}+\varepsilon \gamma \;u^{3}\\ & & =\varepsilon F_{1}\cos\left(\Omega_{d}\tau \right),\ddot{v}+\omega_{2}^{2}v+2\varepsilon \mu_{2}\dot{v}+2\varepsilon \mu_{\mathrm{non}}\dot{v}v^{2}+\varepsilon \beta u^{2}\\ & & \;+\;3\varepsilon \gamma u^{2}v+\varepsilon \gamma \;v^{3}=0. \end{array}$$

Here, *u* and *v* represent the RO and AP coordinates, respectively. The RO mode is directly driven by *F*_1_ cos(Ω*_d_τ*) and contains the cubic stiffness term responsible for Duffing bistability. The AP mode is not directly forced; instead, it is excited by the quadratic coupling term *βu* ^2^ generated by the RO motion. This model therefore captures the essential 1:2 IR pathway from the driven RO mode to the AP mode.

By numerically integrating Equation (1) using the classical fourth‐order Runge–Kutta method, with *u* corresponding to *x*_RO_(*ω_d_*) and *v* corresponding to *x*_AP_(2*ω_d_*), we obtain the simulated response maps shown in Figure 3g,h. The simulations reproduce the main experimental features, including the tick‐like RO jump boundary, the enhanced AP response near the IR transition, and the suppression of the RO high‐amplitude branch in the DBC regime. The numerical model also resolves the unstable transition region, where horizontal oscillatory bands represent the min–max amplitude envelope of quasi‐periodic motions generated through a Hopf bifurcation. In the frequency domain, these quasi‐periodic states correspond to mechanical frequency‐comb generation. The agreement between experiment and simulation confirms that Equation (1) captures both the collapse of Duffing bistability and the associated comb‐like instability induced by strong RO–AP coupling.

**FIGURE 4:**
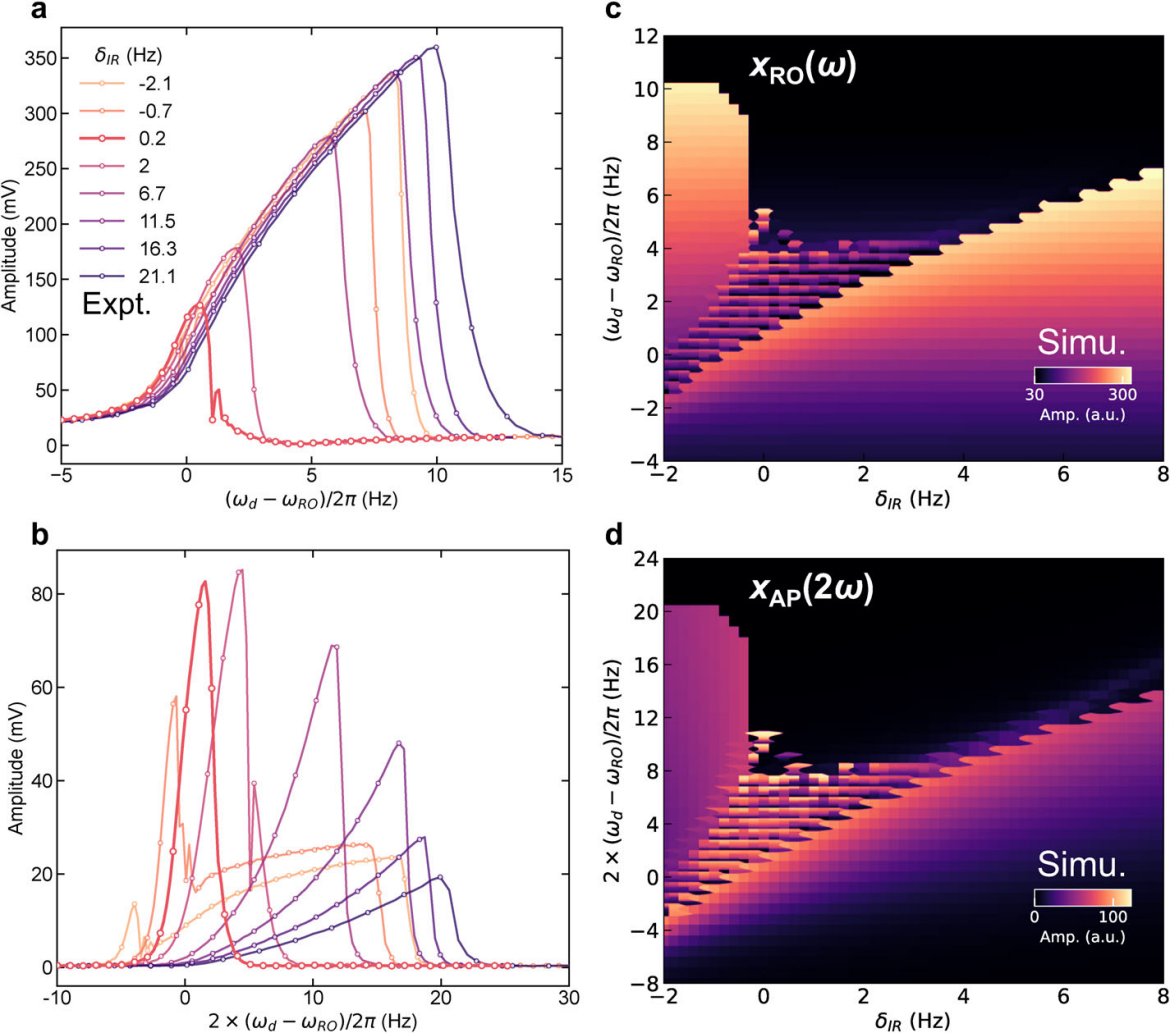
Enhanced DBC under reduced gas damping. (a, b) Experimental nonlinear frequency responses of the RO response *x*_RO_(*ω_d_*) and AP response *x*_AP_(2*ω_d_*) measured at a lower chamber pressure of 10 Pa with *V_a_* = 160 mV. The reduced gas damping increases the quality factor to approximately *Q* ≈ 6000. Compared with the 25 Pa case, the higher‐*Q* condition produces a stronger DBC response: the RO jump frequency shifts by approximately 7.7 Hz from the negative‐IR‐mismatch Duffing branch to the near‐zero‐IR‐mismatch collapsed state. The AP response is simultaneously enhanced near the transition, confirming stronger RO–AP energy transfer. (c, d) Simulated frequency–mismatch response maps of *x*_RO_(*ω_d_*) and *x*_AP_(2*ω_d_*) obtained by reducing the linear damping ratios of both RO and AP modes by a factor of two relative to the baseline simulation (Figure 3g,h). The reduced‐damping simulation reproduces the experimentally observed enhancement of DBC, with the RO jump‐frequency shift increasing from approximately 6.8 Hz in the baseline case to approximately 9.2 Hz. These results show that reducing environmental damping strengthens IR‐mediated energy exchange and amplifies the collapse of the Duffing bistable branch.

To address the role of environmental damping, we further performed comparison experiments at a reduced chamber pressure of 10 Pa, where the quality factor increased to approximately *Q* ≈ 6000. The drive voltage was fixed at *V_a_* = 160 mV to enable direct comparison with the 25 Pa measurements. As shown in Figure 4a,b, DBC remains clearly observable under the higher‐Q condition and becomes more pronounced. In the RO response, the jump frequency decreases by approximately 7.7 Hz between the negative‐IR‐mismatch Duffing‐dominant state and the near‐zero‐IR‐mismatch collapsed state. Under the same drive condition at 25 Pa, the corresponding jump‐frequency reduction is approximately 5.4 Hz (Figure 3e,f). This comparison indicates that reducing gas damping enhances the sensitivity of the Duffing stability boundary to IR‐mediated energy transfer. We note that the jump frequencies in Figure 4a show finite sweep‐to‐sweep variation even away from the near‐zero‐IR‐mismatch transition. This variation is mainly caused by imperfect pressure stabilization at 10 Pa. At this lower pressure, small pressure fluctuations produce noticeable changes in gas damping and therefore in the effective Q factor during different frequency sweeps. Nevertheless, the observed jump‐frequency reduction associated with DBC remains larger than the background sweep‐to‐sweep variation and occurs systematically near the near‐zero IR‐mismatch condition.

The AP response provides a consistent picture. Near the DBC transition, the AP amplitude is strongly enhanced, indicating that more RO energy is transferred into the AP channel when background gas damping is reduced. Therefore, the stronger collapse at 10 Pa is not caused by a change in drive condition, but by the increased modal quality factor, which allows the IR‐mediated energy‐transfer pathway to dominate the nonlinear energy balance more effectively.

We further verified this interpretation using numerical simulations. In the model, the linear damping ratios of both RO and AP modes were reduced by a factor of two while keeping the nonlinear coupling structure unchanged. The simulated colormaps in Figure 4c,d reproduce the experimentally observed enhancement of DBC. To quantify the jump‐frequency reduction, we compare the stable Duffing jump boundary in the negative‐IR‐mismatch regime with that at *δ*_IR_ ≈ 0. The frequency modulation associated with the Hopf‐type bifurcation region is not included in this jump‐frequency estimate. Using this definition, the RO jump‐frequency reduction increases from approximately 6.8 Hz in the baseline simulation (Figure 3g,h) to approximately 9.2 Hz after reducing the linear damping. This agreement between experiment and simulation confirms that DBC is strengthened under higher‐Q operation, because reduced background damping makes the IR‐mediated AP‐channel dissipative pathway more effective in reshaping the Duffing bistability boundary.

If the pressure is further reduced toward 10^−3^ Pa, the Q factors of both RO and AP modes may approach the 10^5^ range, with the remaining losses governed by thermoelastic damping, anchor loss, and material‐related dissipation. The corresponding bandwidths can enter the sub‐Hz regime (potentially below 0.1 Hz), making precise control of *δ*_IR_ ≈ 0 increasingly important. At fixed drive voltage, the higher‐Q RO response can form a stronger nonlinear energy reservoir and transfer more energy into the AP mode; if the AP mode retains appreciable intrinsic damping, the resulting IR‐mediated AP dissipation can enhance the sensitivity of the Duffing stability boundary and preserve a pronounced DBC response. This trend need not increase monotonically, however. If the AP intrinsic damping also becomes extremely small, energy transferred to the AP mode can be stored and coherently exchanged for longer times rather than rapidly dissipated. The system may then show enhanced coherent energy exchange, extended Hopf‐type quasiperiodic modulation, or partial energy return. Accordingly, the DBC window and the critical mismatch may shift or change shape.

### 2.3 Phase‐Resolved Origin of IR Feedback

**FIGURE 5:**
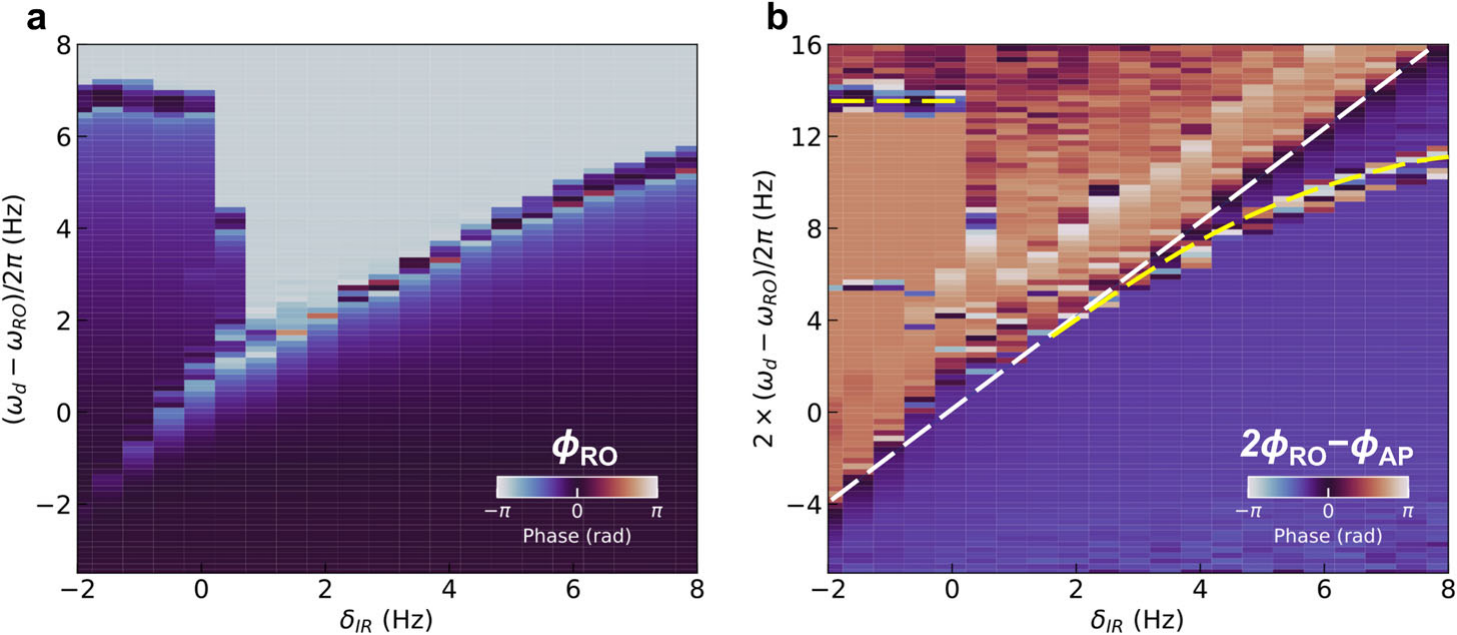
Phase‐resolved signature of IR‐mediated feedback. (a) Experimentally measured phase map of the RO response, *ϕ*_RO_(*ω_d_*), as a function of drive frequency and IR mismatch *δ*_IR_. The phase evolution follows the nonlinear RO response and exhibits an abrupt transition near the Duffing jump boundary, reflecting the collapse of the high‐amplitude branch in the DBC regime. (b) Reconstructed internal phase mismatch, *θ*_IR_ = wrap[2*ϕ*_RO_(*ω_d_*) − *ϕ*_AP_(2*ω_d_*)], which determines the sign and strength of the AP feedback acting on the RO mode. The white dashed line indicates the AP resonance condition, while the yellow dashed line marks the RO jump boundary. Near *δ*_IR_ ≈ 0, strong AP excitation coincides with a dissipative phase condition near the RO resonance, converting the AP mode into the strongest resistive energy‐sink channel and triggering DBC.

To identify the IR Feedback Pathway of AP Mode Responsible for DBC, We Further Measured the phase response of the RO and AP modes, as shown in Figure 5. The phase values were extracted directly from the complex lock‐in outputs. Specifically, the RO complex response was measured at the drive frequency *ω_d_* from the central resonator output, giving *ϕ*_RO_(*ω_d_*) = arg[*X*_RO_(*ω_d_*)]. The AP complex response was measured at the second‐harmonic reference 2*ω_d_* from the projected AP modal coordinate of the two outer resonators, giving *ϕ*_AP_(2*ω_d_*) = arg[*X*_AP_(2*ω_d_*)]. The AP modal coordinate was reconstructed from the complex outer‐resonator signals before taking the phase, rather than by averaging the measured phases. The measured phases were unwrapped along the frequency sweep and then wrapped back to the interval [−*π,π*] for plotting.

Figure 5a shows the RO phase map, *ϕ*_RO_(*ω_d_*), across the IR mismatch *δ*_IR_. The RO phase follows the nonlinear Duffing response and changes abruptly near the jump boundary, reflecting the transition from the high‐amplitude branch to the low‐amplitude branch. However, the RO phase alone only identifies where the Duffing jump occurs; it does not determine how the AP mode feeds back to the RO mode. To resolve this feedback pathway, we reconstruct the internal phase mismatch, *θ*_IR_ = wrap[2*ϕ*_RO_(*ω_d_*) − *ϕ*_AP_(2*ω_d_*)], as shown in Figure 5b. This phase is the experimentally measurable counterpart of the internal phase variable in the slow‐flow model and provides the phase information required to interpret the IR‐mediated feedback (Note S2 Equations S33–S36).

The topology of the *θ*_IR_ colormap provides direct evidence that the AP‐mediated feedback changes character when the response crosses the AP resonance. In Figure 5b, the white dashed line marks the AP resonance condition, and the yellow dashed line marks the RO jump. The AP resonance line approximately separates the phase map into two regions. Below the AP resonance line, *θ*_IR_ is predominantly in the negative‐phase region, whereas above the AP resonance line, *θ*_IR_ shifts to the positive‐phase region. Before the AP resonance is crossed, the AP‐mediated feedback is more strongly projected onto the dissipative quadrature of the RO motion, so the AP channel behaves more like a resistive energy‐removal pathway. After the AP resonance is crossed, the phase of the AP response reverses, and the feedback becomes more reactive or partially energy‐returning with respect to the RO motion.

For negative IR mismatch, the RO high‐amplitude Duffing branch crosses the AP resonance before reaching its final jump boundary. After this crossing, the AP‐mediated feedback on the RO mode lies predominantly in the post‐resonance phase region, where the feedback is more reactive or energy‐returning rather than resistive. As a result, the AP response produces a local perturbation of the Duffing backbone, but the high‐amplitude branch can still continue to a higher jump frequency. This explains why the negative‐mismatch regime retains a broad Duffing response despite observable AP excitation.

Near zero IR mismatch, the phase transition of *θ*_IR_ occurs directly near the RO resonance, and thus the RO branch encounters both strong AP excitation and a dissipative phase projection at the point where it is most sensitive to additional damping. The phase‐weighted AP feedback becomes maximized, the IR‐mediated damping increases sharply, and the high‐amplitude Duffing branch collapses. Thus, the apparent coincidence between the AP resonance region and the RO jump boundary is a consequence of the DBC transition induced by near‐zero IR mismatch. In the Hopf‐type modulation regime, the RO and AP modes undergo intense, alternating energy transfer. The resulting amplitude–phase dynamics give rise to strong intrinsic phase modulation (Figure S6). The simultaneous amplitude and phase modulation provides further evidence that the Hopf‐type response originates from strong bidirectional RO‐AP energy exchange near the DBC transition.

For positive IR mismatch, the RO branch remains largely below the AP resonance line before the jump and therefore experiences AP‐mediated feedback with a continuous dissipative projection. This explains why the RO amplitude and jump frequency remain suppressed. However, because the AP resonance is shifted away from the condition of maximum RO–AP coupling, the AP response and the phase‐weighted feedback do not reach the maximum value observed near zero IR mismatch. Consequently, the system enters an IR‐damped regime rather than the strongest DBC regime.

### 2.4 Energy Collapse and Damping Decomposition

**FIGURE 6:**
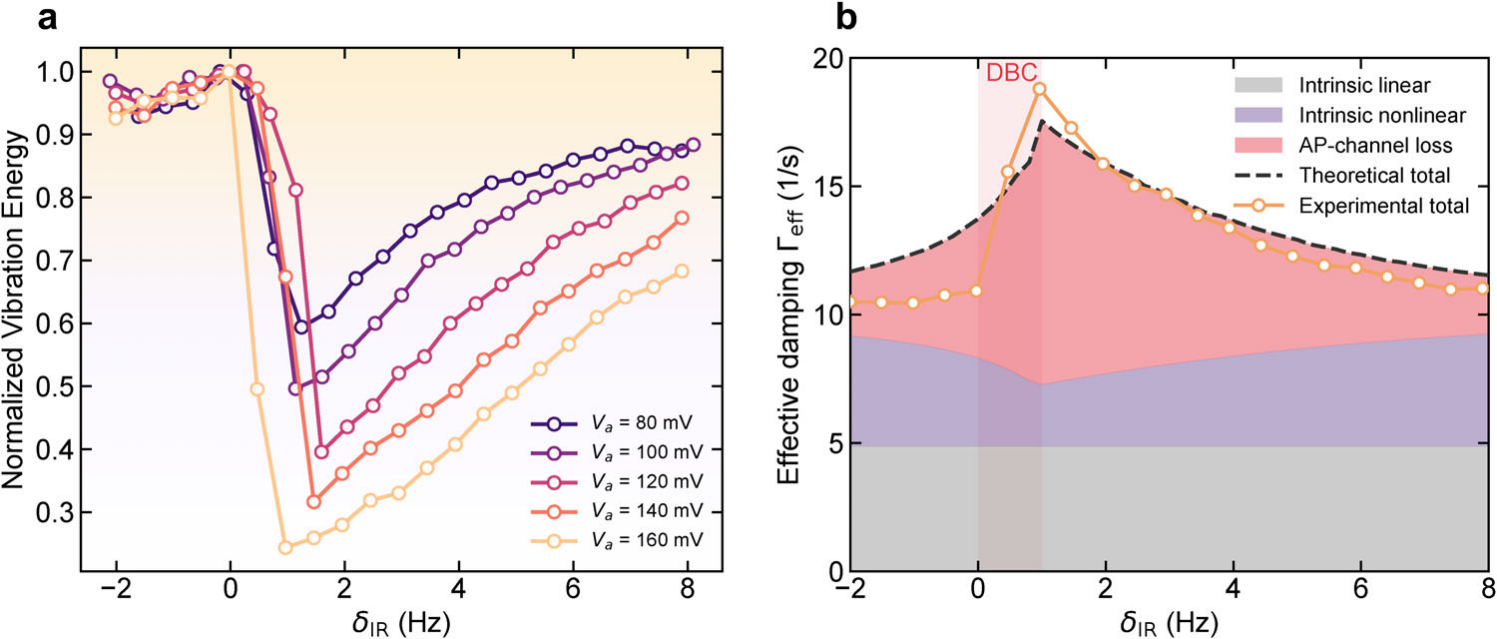
Correlation between system energy collapse and IR‐enhanced damping. (a) Evolution of the normalized total vibration energy, *E*_total_, as a function of IR mismatch *δ*_IR_ for different drive voltages, *V_a_* = 80–160 mV. The energy topology reveals a clear transition near *δ*_IR_ ≈ 0, where the stored vibration energy undergoes a sharp collapse. The energy reduction becomes more pronounced at higher drive voltages, indicating that the DBC transition is strengthened when the RO high‐amplitude Duffing branch stores more energy. (b) Comparison between the experimentally extracted total effective damping rate, Γ_eff_, and the theoretical prediction from the damping decomposition model. The shaded regions show the relative contributions from intrinsic linear damping, intrinsic nonlinear damping, and AP‐channel dissipative contribution. The AP‐channel loss term becomes dominant in the DBC region, producing a peak in Γ_eff_ that coincides with the energy collapse observed in (a).

To quantify the energetic impact of DBC, we introduce the total vibration energy as an order parameter of the coupled system, $E_{\mathrm{total}}=\left(M_{\mathrm{RO}}\omega_{\mathrm{RO}}^{2}x_{\mathrm{RO}}^{2}+M_{\mathrm{AP}}\omega_{\mathrm{AP}}^{2}x_{\mathrm{AP}}^{2}\right)/2$, where *M*_AP_ = 2*M*_RO_ for the two outer resonators. The evolution of *E*_total_ as a function of IR mismatch is shown in Figure 6a. The energy topology exhibits a characteristic tick‐like profile that closely follows the RO jump‐frequency trajectory in Figure 3. This correlation indicates that the stored energy is mainly governed by the RO high‐amplitude branch, while the AP mode provides an additional energy‐transfer pathway through IR coupling. Near the critical IR mismatch, *δ*_IR_ ≈ 1 Hz, the total vibration energy undergoes an abrupt collapse, marking the transition from a stored‐energy Duffing state to a dissipative DBC state. At the highest drive voltage, *V_a_* = 160 mV, the stored energy is reduced by approximately 80% relative to the Duffing‐dominant state. The collapse becomes stronger at higher drive voltages because larger RO oscillation amplitudes enhance the nonlinear RO–AP energy‐transfer rate.

To identify the dissipative mechanism governing the energy collapse, we extracted the total effective damping rate Γ_eff_ directly from the measured RO response at *V_a_* = 160 mV. The extraction is based on the steady‐state power balance of the driven RO mode. For a harmonic response *x*_RO_(*t*)  = *x*_jump_ cos(ω_jump_ *t* − θ_jump_), the cycle‐averaged input power from the external drive is *P*_in_ = *F*_drive_ ω_jump_ *x*_jump_ *sin*θ_jump_/2, while the cycle‐averaged dissipated power associated with an effective damping rate Γ_eff_ is $P_{\mathrm{diss}}=M_{\mathrm{RO}}\;\Gamma_{\mathrm{eff}}\omega_{\mathrm{jump}}^{2}x_{\mathrm{jump}}^{2}/2$. Equating ⟨*P*_in_⟩ and ⟨*P*_diss_⟩ gives

(2) $$\Gamma_{eff}=\frac{F_{drive}\sin\theta_{jump}}{M_{RO}\omega_{jump}x_{jump}}$$

Here, *F*_drive_ is the calibrated electrostatic driving force, *M*_RO_ is the effective mass of the RO mode, and *x*_jump_, *ω*_jump_, and *θ*_jump_ are the RO amplitude, angular frequency, and phase lag relative to the drive, respectively, evaluated on the high‐amplitude branch immediately before the Duffing jump. This procedure provides an experimental estimate of the total damping required to sustain the measured response at the jump boundary.

The calculated Γ_eff_ is compared with the theoretical effective damping rate derived from the steady‐state energy balance in Note S3.3. As given in Equation S40, the theoretical Γ_eff_ can be decomposed into three contributions: intrinsic linear damping, intrinsic nonlinear damping, and an IR‐mediated AP‐channel dissipative contribution. The intrinsic linear damping corresponds to the *µ*_1_ term in the model and provides an approximately constant background loss, which is mainly determined by gas damping under the experimental pressure. The intrinsic nonlinear damping corresponds to the *µ*_non_ term and varies smoothly with the RO oscillation amplitude, representing amplitude‐dependent nonlinear loss of the RO mode. The third contribution originates from the nonlinear RO–AP energy‐transfer pathway activated by the 1:2 IR. Since the AP mode has finite damping, any steady AP excitation requires net power flow from the RO mode into the AP channel; this contribution therefore represents the net AP‐channel loss projected into the effective damping balance.

As shown in Figure 6b, the experimentally extracted total damping agrees well with the theoretical total damping obtained from Equation S40. The decomposition shows that the intrinsic linear damping remains nearly constant as *δ*_IR_ is swept, while the intrinsic nonlinear damping varies only smoothly with the RO amplitude. In contrast, the IR‐mediated AP‐channel dissipative contribution increases abruptly near the DBC transition and becomes the dominant component of Γ_eff_ when *δ*_IR_ approaches the near‐zero regime. Importantly, this damping peak occurs at the same IR mismatch where *E*_total_ collapses in Figure 6a, revealing an inverse correlation between stored vibration energy and effective damping. This correlation shows that DBC is not caused by the fixed background loss or by a gradual increase of intrinsic nonlinear damping, but by the rapid growth of the IR‐mediated AP‐channel loss, which opens an additional dissipative pathway, suppresses the RO high‐amplitude branch, and collapses the Duffing bistability.

## 3 Conclusion

In conclusion, we demonstrate an IR‐mismatch‐controlled collapse of Duffing bistability, termed DBC, in a coupled micromechanical system. Using a serially coupled triple‐resonator architecture, we independently tune the 1:2 IR condition between the RO and AP modes while preserving simultaneous access to both modal responses. When the IR mismatch approaches the near‐zero condition, the AP mode is strongly activated by the nonlinear RO motion and opens an additional IR‐mediated energy‐transfer pathway. This pathway suppresses the high‐amplitude Duffing branch, downshifts the jump frequency, and reduces the stored vibration energy. At *V_a_* = 160 mV, the total vibration energy is depleted by approximately 80% compared with the Duffing‐dominant state, corresponding to an On/Off switching contrast of approximately 21 dB between the high‐amplitude bistable state and the collapsed state.

The experimental maps, numerical simulations, phase response, and damping analysis consistently identify DBC as a dissipation‐driven nonlinear transition rather than a simple amplitude‐saturation effect. The frequency–mismatch maps show that the collapse occurs in a finite mismatch window around the near‐zero IR condition, where the RO jump boundary is strongly reshaped, and the high‐amplitude branch can no longer be sustained. Increasing the drive voltage strengthens the Duffing hardening and enlarges the contrast between the high‐energy Duffing branch and the collapsed state, while the DBC transition remains controlled by the IR mismatch. Additional low‐pressure measurements further show that DBC is enhanced at higher quality factor, indicating that reducing background damping allows the IR‐mediated AP‐channel loss to dominate the nonlinear energy balance more effectively.

A central result of this work is the phase‐resolved identification of the AP‐mediated feedback mechanism. The AP amplitude alone only indicates where the internal mode is excited; it does not determine how the AP response feeds back into the RO dynamics. By reconstructing the internal phase mismatch between the RO response at *ω_d_* and the AP response at 2*ω_d_*, we show that the AP resonance separates two phase domains in the feedback topology. Before and after crossing the AP resonance, the AP‐mediated feedback is projected differently onto the RO quadratures, changing from a more dissipative projection to a more reactive projection. For negative IR mismatch, the RO branch crosses the AP resonance before reaching the final jump boundary, so the AP response produces only a local perturbation and the Duffing branch can survive to higher frequency. Near zero IR mismatch, strong AP excitation and favorable dissipative phase projection occur in the frequency range where the RO branch is most sensitive to additional damping. This maximizes the phase‐weighted IR feedback and triggers the collapse of the high‐amplitude Duffing branch. For positive IR mismatch, the AP‐mediated damping remains present but is no longer maximized, leading to a suppressed Duffing response rather than the strongest DBC.

The effective damping analysis provides quantitative evidence for tunable dissipation. The experimentally extracted Γ_eff_ peaks at the same IR mismatch where the total vibration energy collapses. By decomposing Γ_eff_ into intrinsic linear damping, intrinsic nonlinear damping, and the IR‐mediated AP‐channel dissipative contribution, we show that the sharp damping peak cannot be explained by intrinsic losses alone. The intrinsic linear damping remains nearly constant, and the intrinsic nonlinear damping varies only smoothly with RO amplitude. In contrast, the AP‐channel dissipative contribution increases abruptly near the DBC transition and becomes the dominant component of the total effective damping. This decomposition directly links DBC to the growth of an IR‐mediated dissipative pathway and shows that the dissipation landscape is actively reshaped by tuning the IR mismatch.

These results establish DBC as a mechanism for dissipation engineering in nonlinear multimode resonators. Unlike conventional Duffing switching or mechanical memory schemes that rely mainly on changing drive amplitude within a fixed dissipation landscape, our platform uses the IR mismatch *δ*_IR_ as an independent control axis. This enables a “hold‐and‐collapse” operation: the device can sustain a high‐amplitude Duffing state under negative IR mismatch and then collapse it by activating the AP‐mediated dissipative channel near the IR condition, without requiring a large change in drive power. This provides a route toward high‐contrast mechanical switching, amplitude limiting, and reconfigurable nonlinear filtering, where the effective cutoff and stability boundary can be programmed through modal frequency tuning.

More broadly, the present work converts IR‐induced nonlinear damping from a phenomenological amplitude‐dependent loss into a phase‐resolved and mismatch‐controlled feedback mechanism. The same principle may be extended to other coupled nonlinear systems in which a resonant internal channel can be tuned to reshape the effective dissipation and nonlinear stability boundary. In this sense, DBC provides an experimentally accessible platform for studying controllable nonlinear dissipation, modal energy transfer, and stability engineering in MEMS and NEMS resonators, with potential relevance to mechanical signal processing, robust sensing, and multimode phononic control.

## Author Contributions

**J.C**.: conceptualization, data curation, formal analysis, investigation, methodology, software, validation, visualization, writing – original draft, writing – review and editing, funding acquisition. **X.Z**.: conceptualization, formal analysis, investigation, software, and writing – review and editing. **T.T**.: investigation, methodology, project administration, resources, funding acquisition, supervision, and writing – review and editing. **N.W**.: investigation and writing – review and editing. **S.T**.: project administration, resources, funding acquisition, supervision, and writing – review and editing.

## Conflicts of Interest

The authors declare no conflicts of interest.

## Supporting information




**Supporting File**: advs77439‐0001‐SuppMat.pdf.

## Data Availability

The data that support the findings of this study are available on request from the corresponding author. The data are not publicly available due to privacy or ethical restrictions.
