# Supplementary material for: Internal Resonance Mismatch‐Controlled Dissipative Bistability Collapse in Coupled Micromechanical Oscillators
Source: Adv Sci (Weinh). 2026 Aug 30:e77439. Online ahead of print. doi: 10.1002/advs.77439 (PMC13526701; doi:10.1002/advs.77439)
Supplement: Supplementary file 1 [file ADVS-9999-e77439-s001.pdf]

Supplementary Information

Internal-Resonance-Mismatch-Controlled Dissipative Bistability Collapse in Coupled Micromechanical Oscillators

---

SUPPLEMENTARY NOTE I. Experimental setup

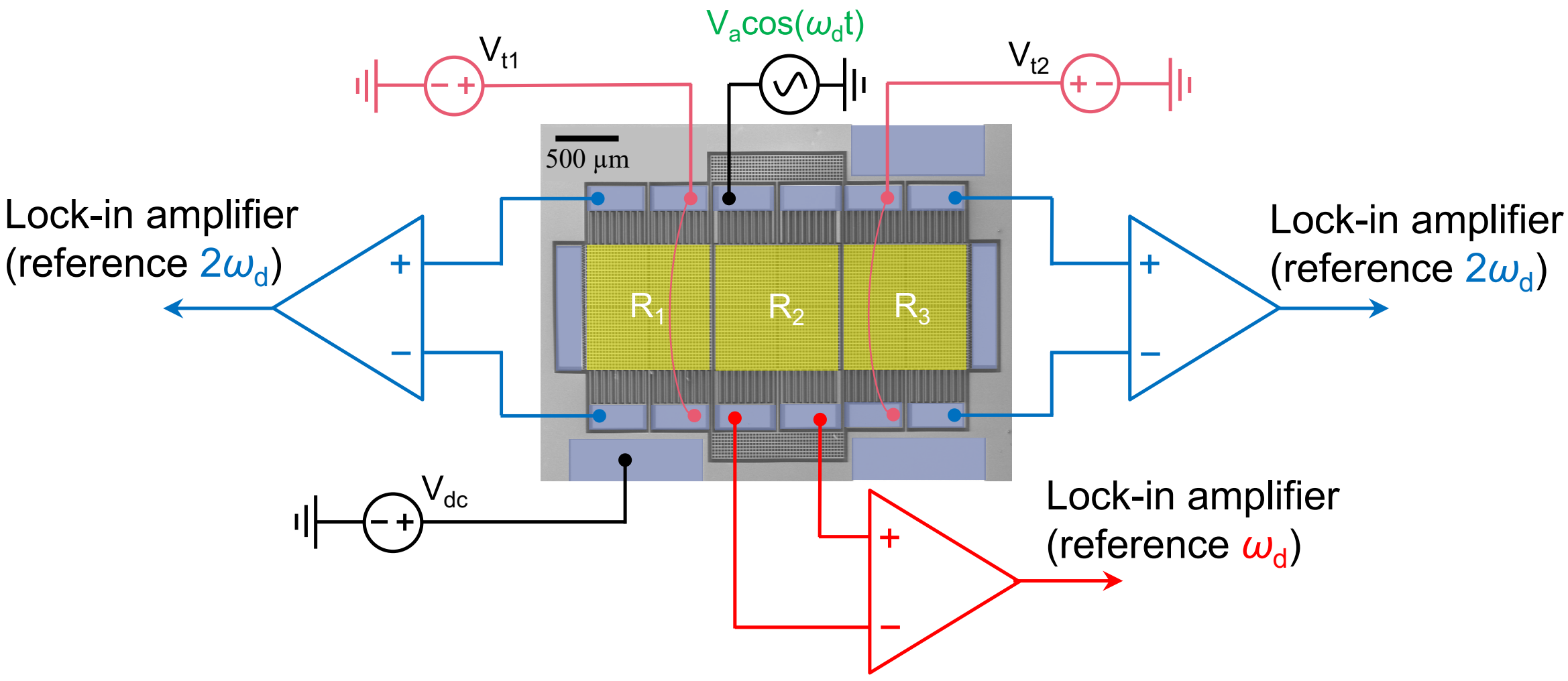

Figure S1: False-color SEM micrograph of the serially coupled triple-resonator device and the experimental setup schematic.

The device was measured in 25 Pa vacuum chamber and the Q-factor of RO and AP mode in linear regime were about 3000 limited by the air damping. The DC bias 10 V was applied on the proof mass and the AC driving signal V_a applied on the parallel plates electrode of the center resonator R_2. Two tuning DC bias were applied on the R_1 and R_3 respectively for compensating the stiffness mismatch and electrostatic tuning the AP mode frequency. The RO mode motion was mainly from R_2 therefore the resonant signal of RO mode was assumed as the output of R_2 from the pair of differential parallel plate electrodes of R_2 and the signal demodulation frequency is ω_d. On the other hand the AP mode motion concentrates on the two outer resonators, therefore the R_1 output signal from left side parallel plate electrodes demodulated by $2\omega_d$ is assumed as the resonant signal of AP mode under balance motion. The frequency sweeping measurements were done by using the lock in amplifier (The Zurich Instruments UHFLI).

When DC biases of 50.5 V and 60 V were applied to the tuning electrodes of R_1 and R_3, respectively, the frequency ratio of the AP mode ω_{AP} to the RO mode ω_{RO} was tuned to approximately 2:1. At the same time, the stiffness mismatch between R_1 and R_3 was compensated, resulting

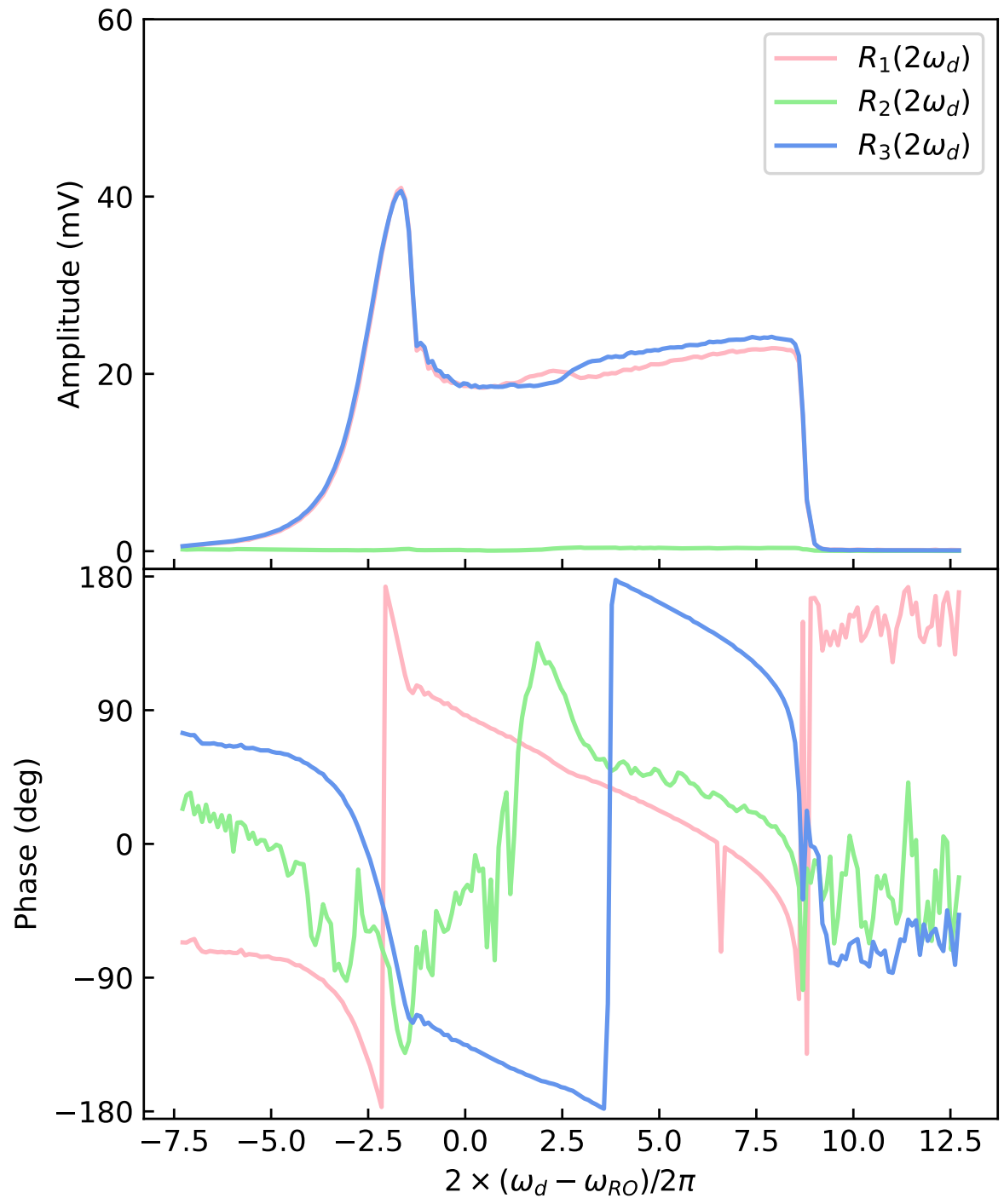

Figure S2: The amplitude and phase of superhamonic output under IR.

in balanced motion of the two outer resonators. When the RO mode was driven into the nonlinear regime with an AC voltage of 160 mV, the superharmonic responses $R_1(2\omega)$, $R_2(2\omega)$, and $R_3(2\omega)$ were measured and are plotted in Fig. S2. The signals $R_1(2\omega)$ and $R_3(2\omega)$ exhibit nearly equal amplitudes in anti-phase, while $R_2(2\omega)$ remains nearly static, indicating that structural asymmetries were compensated and the structure operated in a balanced state between the two outer resonators. The pronounced superharmonic components $R_1(2\omega)$ and $R_3(2\omega)$ confirm that the AP mode was successfully excited via 1:2 IR with the RO mode.

SUPPLEMENTARY NOTE II. Theoretical derivation of dynamic equations

The nonlinear dynamic between RO and AP modes are described by (S1) and (S2) respectively. The nonlinear coupling force consists of a quadratic term $\beta(x_{RO} - x_{AP})^2$ and a cubic term $\gamma(x_{RO} - x_{AP})^3$. $\beta(x_{RO} - x_{AP})^2$ exerts a negative effect on the duffing frequency since the energy transfer to the AP mode. $\gamma(x_{RO} - x_{AP})^3$ exerts a positive effect on the frequency since the nonlinear hardening effect. On the other hand, both $\beta(x_{RO} - x_{AP})^2$ and $\gamma(x_{RO} - x_{AP})^3$ exert positive effect on the frequency since AP mode gain energy from RO mode from square nonlinear coupling and stiffness hardens under cubic nonlinear coupling. The equations of motion can be described as follows:

$$M\ddot{x}_{RO}+k_{RO}x_{RO}+r_{RO}\dot{x}_{RO}+r_{non}x_{RO}^2\dot{x}_{RO}-c(x_{RO}-x_{AP})+d(x_{RO}-x_{AP})^3 = F_0\cos\Omega_0 t \quad \text{(S1)}$$

$$M\ddot{x}_{AP}+k_{AP}x_{AP}+r_{AP}\dot{x}_{AP}+r_{non}x_{AP}^2\dot{x}_{AP}+c(x_{RO}-x_{AP})+d(x_{RO}-x_{AP})^3 = 0 \quad \text{(S2)}$$

where x_{RO} and x_{AP} represent the displacement amplitudes of the RO and AP modes, respectively. The effective mass of each resonator is assumed to be identical and is denoted by M. The parameters k_{RO} and k_{AP} define the effective linear stiffness for each mode. To satisfy the conditions for 1:2 internal resonance, these stiffnesses are related by $k_{AP} \approx 4k_{RO}$. Energy dissipation within the system is captured by the linear damping coefficients r_{RO} and r_{AP}, as well as a nonlinear damping coefficient r_{non} that accounts for amplitude-dependent losses. The nonlinear inter-modal coupling is mediated by stiffness terms, where c and d denote the quadratic and cubic nonlinear stiffness coefficients, respectively. Finally, the system is externally excited via the RO mode by a driving force with amplitude F_0 and angular frequency Ω_0. The expanded form of the equation of motion can be expressed as:

$$\begin{gathered} M\ddot{x}_{RO}+k_{RO}x_{RO}+r_{RO}\dot{x}_{RO}+r_{non}x_{RO}^2\dot{x}_{RO} \\ -c(x_{AP}^2-2x_{RO}x_{AP}+x_{RO}^2)+d(x_{AP}^3-3x_{RO}x_{AP}^2+3x_{RO}^2x_{AP}-x_{RO}^3) = F_0\cos\Omega_0 t \quad \text{(S3)} \\ M\ddot{x}_{AP}+k_{AP}x_{AP}+r_{AP}\dot{x}_{AP}+r_{non}x_{AP}^2\dot{x}_{AP} \\ +c(x_{AP}^2-2x_{RO}x_{AP}+x_{RO}^2)+d(x_{AP}^3-3x_{RO}x_{AP}^2+3x_{RO}^2x_{AP}-x_{RO}^3) = 0 \quad \text{(S4)} \end{gathered}$$

The excitation of internal resonance, manifested through intermodal energy transfer and secular terms, requires precise matching between the frequency combinations arising from nonlinear terms and the system's eigenfrequencies (or their integer linear combinations). In the absence of such matching conditions, the contribution of these nonlinear terms to the long-term system dynamics becomes insignificant and may be disregarded. The frequency composition of the nonlinear terms in Eq. (S3) and Eq. (S4) is examined below:

The linear solution of x_{RO} is given by $x_{RO} = A_x e^{i\omega_{RO}t} + A_x e^{-i\omega_{RO}t}$. The linear solution of x_{AP} is given by $x_{AP} = A_y e^{i\omega_{AP}t} + A_y e^{-i\omega_{AP}t}$.

- x_{RO}^2 produces $e^{i2\omega_{RO}t}$ and constant component.
- $x_{RO}x_{AP}$ produces $e^{i(\omega_{AP}-\omega_{RO})t} = e^{i\omega_{RO}t}$ and $e^{i(\omega_{AP}+\omega_{RO})t} = e^{i3\omega_{RO}t}$.
- x_{AP}^2 produces $e^{i2\omega_{AP}t}$ and constant component..

- x_{RO}^3 produces $e^{i3\omega_{RO}t}$ and $e^{i\omega_{RO}t}$.
- $x_{RO}x_{AP}^2$ produces $e^{i(2\omega_{AP}+\omega_{RO})t} = e^{i5\omega_{RO}t}$ and $e^{i\omega_{RO}t}$.
- $x_{RO}^2 x_{AP}$ produces $e^{i(\omega_{AP}+2\omega_{RO})t} = e^{i4\omega_{RO}t}$ and $e^{i\omega_{AP}t}$.
- x_{AP}^3 produces $e^{i3\omega_{AP}t} = e^{i6\omega_{RO}t}$ and $e^{i\omega_{AP}t} = e^{i2\omega_{RO}t}$.

For the x_{RO}-direction dynamics (Eq. S3), only nonlinear terms ($x_{RO}x_{AP}$, x_{RO}^3, $x_{RO}x_{AP}^2$) containing the $e^{i\omega_{RO}t}$ component contribute significantly to the long-term system behavior. Similarly, for the x_{AP}-direction dynamics (Eq. S4), only terms (x_{RO}^2, $x_{RO}^2 x_{AP}$, and x_{AP}^3) with the $e^{i\omega_{AP}t} = e^{i2\omega_{RO}t}$ component affect the long-term evolution. After eliminating negligible terms that have minimal impact on the system dynamics, we obtain the following reduced equations:

$$M\ddot{x}_{RO} + k_{RO}x_{RO} + r_{RO}\dot{x}_{RO} + r_{non}x_{RO}^2\dot{x}_{RO} + 2cx_{RO}x_{AP} + 3dx_{RO}x_{AP}^2 + dx_{RO}^3 = F_0\cos\Omega_0 t \tag{S5}$$

$$M\ddot{x}_{AP} + k_{AP}x_{AP} + r_{AP}\dot{x}_{AP} + r_{non}x_{AP}^2\dot{x}_{AP} + cx_{RO}^2 + 3dx_{RO}^2 x_{AP} + dx_{AP}^3 = 0 \tag{S6}$$

To study the coupled motion more generally, the eigenfrequency of RO mode is normalized to 2π rad/s by using a new time scale $\tau = \sqrt{k_{RO}/4\pi^2 M}t$ (differential transformation are $\frac{d}{dt} = \sqrt{\frac{k_{RO}}{4\pi^2 M}}\frac{d}{d\tau}$, $\frac{d^2}{dt^2} = \frac{k_{RO}}{4\pi^2 M}\frac{d^2}{d\tau^2}$), then the nonlinear dynamic equation is established:

$$\begin{aligned}\frac{k_{RO}}{4\pi^2}\ddot{x}_{RO} + k_{RO}x_{RO} + r_{RO}\sqrt{\frac{k_{RO}}{4\pi^2 M}}\dot{x}_{RO} + r_{non}\sqrt{\frac{k_{RO}}{4\pi^2 M}}\dot{x}_{RO}x_{RO}^2 \\ + 2cx_{RO}x_{AP} + 3dx_{RO}x_{AP}^2 + dx_{RO}^3 = F_0\cos\Omega_0\sqrt{\frac{4\pi^2 M}{k_{RO}}}\tau\end{aligned} \tag{S7}$$

$$\frac{k_{RO}}{4\pi^2}\ddot{x}_{AP} + k_{AP}x_{AP} + r_{AP}\sqrt{\frac{k_{RO}}{4\pi^2 M}}\dot{x}_{AP} + r_{non}\sqrt{\frac{k_{RO}}{4\pi^2 M}}\dot{x}_{AP}x_{AP}^2 + cx_{RO}^2 + 3dx_{RO}^2 x_{AP} + dx_{AP}^3 = 0 \tag{S8}$$

Eq. (S7) and (S8) divide both sides of the equal sign by $\frac{k_{RO}}{4\pi^2}$ to get Eq. (9), and Eq. (10).

$$\ddot{x}_{RO} + 4\pi^2 x_{RO} + \frac{2\pi r_{RO}}{k_{RO}}\sqrt{\frac{k_{RO}}{M}}\dot{x}_{RO} + \frac{2\pi r_{non}}{k_{RO}}\sqrt{\frac{k_{RO}}{M}}\dot{x}_{RO}x_{RO}^2 + \frac{8\pi^2 c}{k_{RO}}x_{RO}x_{AP} + \frac{12\pi^2 d}{k_{RO}}x_{RO}x_{AP}^2 + \frac{4\pi^2 d}{k_{RO}}x_{RO}^3 = \frac{4\pi^2 F_0}{k_{RO}}\cos\Omega_0\sqrt{\frac{4\pi^2 M}{k_{RO}}}\tau \tag{S9}$$

$$\ddot{x}_{AP} + \frac{4\pi^2 k_{AP}}{k_{RO}}x_{AP} + \frac{2\pi r_{AP}}{k_{RO}}\sqrt{\frac{k_{RO}}{M}}\dot{x}_{AP} + \frac{2\pi r_{non}}{k_{RO}}\sqrt{\frac{k_{RO}}{M}}\dot{x}_{AP}x_{AP}^2 + \frac{4\pi^2 c}{k_{RO}}x_{RO}^2 + \frac{12\pi^2 d}{k_{RO}}x_{RO}^2 x_{AP} + \frac{4\pi^2 d}{k_{RO}}x_{AP}^3 = 0 \tag{S10}$$

By parameterizing the Eq. (S9) and Eq. (S10) with the respective parameters ($u = x_{RO}, v = x_{AP}, \omega_1 = 2\pi, \omega_2 = 2\pi\sqrt{k_{AP}/k_{RO}}, \mu_1 = (\pi r_{RO}/\epsilon k_{RO})\sqrt{k_{RO}/M}, \mu_{non} = (\pi r_{non}/\epsilon k_{RO})\sqrt{k_{RO}/M}, \mu_2 = (\pi r_{AP}/\epsilon k_{RO})\sqrt{k_{RO}/M}, \epsilon = 0.001, \beta = 4\pi^2 c/\epsilon k_{RO}, \gamma = 4\pi^2 d/\epsilon k_{RO}, F_1 = 4\pi^2 F_0/\epsilon k_{RO}$, and $\Omega_d = \sqrt{4\pi^2 M/k_{RO}}\Omega_0$), we obtain the resulting Eq. (11) and Eq. (12).

$$\ddot{u} + \omega_1^2 u + 2\epsilon\mu_1\dot{u} + 2\epsilon\mu_{non}u^2\dot{u} + 2\epsilon\beta uv + 3\epsilon\gamma uv^2 + \epsilon\gamma u^3 = \epsilon F_1\cos\Omega_d\tau \tag{S11}$$

$$\ddot{v} + \omega_2^2 v + 2\epsilon\mu_2\dot{v} + 2\epsilon\mu_{non}v^2\dot{v} + \epsilon\beta u^2 + 3\epsilon\gamma u^2 v + \epsilon\gamma v^3 = 0 \tag{S12}$$

The perturbation method of multiple scales is used to study the qualitative behavior of the system. The approximate solution of Eq. (S11) and Eq. (S12) are expressed as a function of two-time scales:

$$u(t,\epsilon) = u_0(T_0,T_1) + \epsilon u_1(T_0,T_1) + o(\epsilon^2), \tag{S13}$$
$$v(t,\epsilon) = v_0(T_0,T_1) + \epsilon v_1(T_0,T_1) + o(\epsilon^2), \tag{S14}$$

where $T_0 = \tau$ and $T_1 = \epsilon\tau$, using chain rules and dropping orders higher than one, time derivatives are

$$\frac{d}{dt} = D_0 + \epsilon D_1 + o(\epsilon^2), \tag{S15}$$
$$\frac{d^2}{dt^2} = D_0^2 + 2\epsilon D_0 D_1 + o(\epsilon^2). \tag{S16}$$

where $D_0 = \partial/\partial T_0$ and $D_1 = \partial/\partial T_1$. Substitute Eq. (S13-S14) into Eq. (S11) and Eq. (S12) to get:

Equating the components of order ϵ^0 and ϵ^1 lead to:

Order ϵ^0:

$$D_0^2 u_0 + \omega_1^2 u_0 = 0, \tag{S17}$$
$$D_0^2 v_0 + \omega_2^2 v_0 = 0, \tag{S18}$$

Order ϵ^1:

$$D_0^2 u_1 + \omega_{RO}^2 u_1 = -2D_0 D_1 u_0 - 2\mu_1 D_0 u_0 - 2\mu_{non} D_0 u_0 u_0^2 - 2\beta u_0 v_0 - 3\gamma u_0 v_0^2 - \gamma u_0^3 + F_1 \cos\Omega_d \tau, \tag{S19}$$

$$D_0^2 v_1 + \omega_{AP}^2 v_1 = -2D_0 D_1 v_0 - 2\mu_1 D_0 v_0 - 2\mu_{non} D_0 v_0 v_0^2 - \beta u_0^2 - 3\gamma u_0^2 v_0 - \gamma v_0^3. \tag{S20}$$

The solutions of Eq. (S19) and Eq. (S20) are expressed as

$$u_0 = A_u(T_1)\cos(\omega_1 T_0 + \phi_u) = A_u(T_1)e^{i\omega_1 T_0} + \bar{A}_u(T_1)e^{-i\omega_1 T_0}, \tag{S21}$$
$$v_0 = A_v(T_1)\cos(\omega_2 T_0 + \phi_v) = A_v(T_1)e^{i\omega_2 T_0} + \bar{A}_v(T_1)e^{-i\omega_2 T_0}, \tag{S22}$$

where $A_u = \frac{1}{2}a_u e^{i\phi_u}$, $A_v = \frac{1}{2}a_v e^{i\phi_v}$, $\bar{A}_u = \frac{1}{2}a_u e^{-i\phi_u}$, $\bar{A}_v = \frac{1}{2}a_v e^{-i\phi_v}$; a_u and a_v are the real part of the first order approximate amplitude A_1 and A_2 respectively; ϕ_u, ϕ_v are the phase difference between u and actuation force and the phase difference between v and actuation force respectively, and $A_u(T_1)$ and $A_v(T_1)$ are undetermined amplitudes. The tuning parameters σ and σ_1 to are used to describe the level of internal and external resonances, respectively. Ω_d and ω_2 can be expressed as,

$$\Omega_d = \omega_1 + \epsilon\sigma, \tag{S23}$$
$$\omega_2 = 2\omega_1 + \epsilon\sigma_1, \tag{S24}$$

Taking Eq. (S23-S24) into Eq. (S21) and Eq. (S22), we can obtain:

$$
\begin{aligned}
D_0^2 u_1 + \omega_1^2 u_1 = &\\
&\Big(-2i\omega_1 D_1 A_u - 2i\omega_1 \mu_1 A_u - 2i\omega_1 \mu_{non} A_u A_u \bar{A}_u - 2\beta \bar{A}_u A_v e^{i\sigma_1 T_1} \\
&\qquad -6\gamma A_u A_v \bar{A}_v - 3\gamma A_u^2 \bar{A}_u + \frac{1}{2} M_1 e^{i\sigma T_0}\Big) e^{i\omega_1 T_1} \\
&+ \Big(2i\omega_1 D_1 \bar{A}_u + 2i\omega_1 \mu_1 \bar{A}_u + 2i\omega_1 \mu_{non} \bar{A}_u A_u \bar{A}_u - 2\beta A_u \bar{A}_v e^{-i\sigma_1 T_1} \\
&\qquad -6\gamma \bar{A}_u A_v \bar{A}_v - 3\gamma A_u \bar{A}_u^2 + \frac{1}{2} M_1 e^{-i\sigma T_1}\Big) e^{-i\omega_1 T_0} \\
&- 2i\omega_1 \mu_{non} (A_u^3 e^{i3\omega_1 T_0} + \bar{A}_u^3 e^{-i3\omega_1 T_0}) \\
&- 3\gamma (A_u A_v^2 e^{i(5\omega_1 + 2\epsilon\sigma_1)T_0} + \bar{A}_u \bar{A}_v^2 e^{-i(5\omega_1 + 2\epsilon\sigma_1)T_0} + A_u \bar{A}_v^2 e^{i(3\omega_1 + 2\epsilon\sigma_1)T_0} + \bar{A}_u A_v^2 e^{-i(5\omega_1 - 2\epsilon\sigma_1)T_0}) \\
&- \gamma (A_u^3 e^{i3\omega_1 T_0} + \bar{A}_u^3 e^{-i3\omega_1 T_0}) \\
&- 2\beta (A_u A_v e^{i(3\omega_1 + \epsilon\sigma_1)T_0} + \bar{A}_u \bar{A}_v e^{-i(3\omega_1 + \epsilon\sigma_1)T_0})
\end{aligned} \tag{S25}
$$

$$
\begin{aligned}
D_0^2 v_1 + \omega_2^2 v_1 = &\\
&\big(-2i\omega_2 D_1 A_v - 2i\omega_2 \mu_2 A_v - 2i\omega_2 \mu_{non} A_v A_v \bar{A}_v - \beta A_u^2 e^{-i\sigma_1 T_1} \\
&\qquad -6\gamma A_u \bar{A}_u A_v - 3\gamma A_v^2 \bar{A}_v\big) e^{i\omega_2 T_0} \\
&+ \big(2i\omega_2 D_1 \bar{A}_v + 2i\omega_2 \mu_2 \bar{A}_v + 2i\omega_2 \mu_{non} \bar{A}_v A_v \bar{A}_v - \beta \bar{A}_u^2 e^{i\sigma_1 T_1} \\
&\qquad -6\gamma A_u \bar{A}_u \bar{A}_v - 3\gamma A_v \bar{A}_v^2\big) e^{-i\omega_2 T_0} \\
&- 2i\omega_2 \mu_{non} (A_v^3 e^{i3\omega_2 T_0} + \bar{A}_v^3 e^{-i3\omega_2 T_0}) \\
&- 3\gamma (A_u^2 A_v e^{i(2\omega_2 - \epsilon\sigma_1)T_0} + \bar{A}_u^2 \bar{A}_v e^{-i(2\omega_2 - \epsilon\sigma_1)T_0} + A_u^2 \bar{A}_v e^{-i\sigma_1 T_1} + \bar{A}_u^2 A_v e^{i\sigma_1 T_1}) \\
&- \gamma (A_v^3 e^{i3\omega_2 T_0} + \bar{A}_v^3 e^{-i3\omega_2 T_0}) - 2\beta A_u \bar{A}_u
\end{aligned} \tag{S26}
$$

Let the coefficient of the permanent term ($e^{i\omega_1 T_0}, e^{i\omega_2 T_0}$) in Eq. (S25) and Eq. (S26) be equal to zero, and get Eq. (S27) and Eq. (S28).

$$
-2i\omega_1 (D_1 A_u + \mu_1 A_u + \mu_{non} A_u^2 \bar{A}_u) - 2\beta \bar{A}_u A_v e^{i\sigma_1 T_1} - 6\gamma A_u A_v \bar{A}_v - 3\gamma A_u^2 \bar{A}_u + \frac{1}{2} M_1 e^{i\sigma T_1} = 0 \tag{S27}
$$

$$
-2i\omega_2 (D_1 A_v + \mu_2 A_v + \mu_{non} A_v^2 \bar{A}_v) - \beta A_u^2 e^{-i\sigma_1 T_1} - 6\gamma A_u \bar{A}_u A_v - 3\gamma A_v^2 \bar{A}_v = 0 \tag{S28}
$$

Taking $A_u = \frac{1}{2} a_u e^{i\phi_u}, A_v = \frac{1}{2} a_v e^{i\phi_v}, \bar{A}_u = \frac{1}{2} a_u e^{-i\phi_u}, \bar{A}_v = \frac{1}{2} a_v e^{-i\phi_v}$ into Eq. (S27) and

Eq.(S28), we can obtain:

$$
\begin{aligned}
&- 2i\omega_1 \left(\frac{1}{2}\dot{a}_u + \frac{1}{2}a_u i\dot{\phi}_u + \frac{1}{2}a_u\mu_1 + \frac{1}{8}a_u^3\mu_{non}\right) e^{i\phi_u} \\
&- \frac{1}{2}\beta a_u a_v e^{i(\sigma_1 T_1+\phi_v-\phi_u)} - \frac{3}{4}\gamma a_u a_v^2 e^{i\phi_u} - \frac{3}{8}\gamma a_u^3 e^{i\phi_u} + \frac{1}{2}F_1 e^{i\sigma T_1} \\
&= (-i\omega_1\dot{a}_u + \omega_1 a_u\dot{\phi}_u - i\omega_1 a_u\mu_1 - \frac{1}{4}i\omega_1 a_u^3\mu_{non} \\
&- \frac{1}{2}\beta a_u a_v e^{i(\sigma_1 T_1+\phi_v-2\phi_u)} - \frac{3}{4}\gamma a_u a_v^2 - \frac{3}{8}\gamma a_u^3 + \frac{1}{2}M_1 e^{i(\sigma T_1-\phi_u)})e^{i\phi_u} = 0
\end{aligned} \tag{S29}
$$

$$
\begin{aligned}
&- 2i\omega_2 \left(\frac{1}{2}\dot{a}_v + \frac{1}{2}a_v i\dot{\phi}_v + \frac{1}{2}a_v\mu_2 + \frac{1}{8}a_v^3\mu_{non}\right) e^{i\phi_v} \\
&- \frac{1}{4}\beta a_u^2 e^{i(2\phi_u-\sigma_1 T_1)} - \frac{3}{4}\gamma a_u^2 a_v e^{i\phi_v} - \frac{3}{8}\gamma a_v^3 e^{i\phi_v} \\
&= (-i\omega_2\dot{a}_v + \omega_2 a_v\dot{\phi}_v - i\omega_2 a_v\mu_2 - \frac{1}{4}i\omega_2 a_v^3\mu_{non} \\
&- \frac{1}{4}\beta a_u^2 e^{i(2\phi_u-\sigma_1 T_1-\phi_v)} - \frac{3}{4}\gamma a_u^2 a_v - \frac{3}{8}\gamma a_v^3)e^{i\phi_v} = 0
\end{aligned} \tag{S30}
$$

By parameterizing the Eq. (S29) and Eq. (S30) with the respective parameters ($\theta_1 = 2\phi_u - \phi_v - \sigma_1 T_1, \theta_2 = \phi_u - \sigma T_1$), when the motion of the two modes reach a stable state, $\partial\theta_1/\partial T_1 = 0, \partial\theta_2/\partial T_1 = 0, \partial a_u/\partial T_1 = 0$, and $\partial a_v/\partial T_1 = 0$, we can obtain:

$$
\begin{aligned}
&- i\omega_1\dot{a}_u + \omega_1 a_u\sigma - i\omega_1 a_u\mu_1 - \frac{1}{4}i\omega_1 a_u^3\mu_{non} - \frac{1}{2}\beta a_u a_v e^{-i\theta_1} - \frac{3}{4}\gamma a_u a_v^2 - \frac{3}{8}\gamma a_u^3 + \frac{1}{2}F_1 e^{-i\theta_2} \\
&= -i\omega_1\dot{a}_u + \omega_1 a_u\sigma - i\omega_1 a_u\mu_1 - \frac{1}{4}i\omega_1 a_u^3\mu_{non} - \frac{3}{4}\gamma a_u a_v^2 - \frac{3}{8}\gamma a_u^3 \\
&- \frac{1}{2}\beta a_u a_v(\cos\theta_1 - i\sin\theta_1) + \frac{1}{2}F_1(\cos\theta_2 - i\sin\theta_2) \\
&= \left(\omega_1 a_u\sigma - \frac{3}{4}\gamma a_u a_v^2 - \frac{3}{8}\gamma a_u^3 - \frac{1}{2}\beta a_u a_v\cos\theta_1 + \frac{1}{2}F_1\cos\theta_2\right) \\
&- i\left(\omega_1\dot{a}_u + \omega_1 a_u\mu_1 + \frac{1}{4}\omega_1 a_u^3\mu_{non} - \frac{1}{2}\beta a_u a_v\sin\theta_1 + \frac{1}{2}F_1\sin\theta_2\right) = 0
\end{aligned} \tag{S31}
$$

$$
\begin{aligned}
&- i\omega_2\dot{a}_v + \omega_2 a_v(2\sigma - \sigma_1) - i\omega_2 a_v\mu_2 - \frac{1}{4}i\omega_2 a_v^3\mu_{non} - \frac{1}{4}\beta a_u^2 e^{i\theta_1} - \frac{3}{4}\gamma a_u^2 a_v - \frac{3}{8}\gamma a_v^3 \\
&= -i\omega_2\dot{a}_v + \omega_2 a_v(2\sigma - \sigma_1) - i\omega_2 a_v\mu_2 - \frac{1}{4}i\omega_2 a_v^3\mu_{non} \\
&- \frac{1}{4}\beta a_u^2(\cos\theta_1 + i\sin\theta_1) - \frac{3}{4}\gamma a_u^2 a_v - \frac{3}{8}\gamma a_v^3 \\
&= \left(\omega_2 a_v(2\sigma - \sigma_1) - \frac{1}{4}\beta a_u^2\cos\theta_1 - \frac{3}{4}\gamma a_u^2 a_v - \frac{3}{8}\gamma a_v^3\right) \\
&- i\left(\omega_2\dot{a}_v + \omega_2 a_v\mu_{AP} + \frac{1}{4}\omega_2 a_v^3\mu_{non} + \frac{1}{4}\beta a_u^2\sin\theta_1\right) = 0
\end{aligned} \tag{S32}
$$

Separating the equation into real and imaginary components yield. The follow averaged equations can be obtained:

$$\omega_1 a_u \sigma - \frac{1}{2}\beta a_u a_v \cos\theta_1 - \frac{3}{4}\gamma a_u a_v^2 - \frac{3}{8}\gamma a_u^3 + \frac{1}{2}F_1 \cos\theta_2 = 0 \tag{S33}$$

$$\omega_1 a_u \mu_1 + \frac{1}{4}\omega_1 a_u^3 \mu_{non} - \frac{1}{2}\beta a_u a_v \sin\theta_1 + \frac{1}{2}F_1 \sin\theta_2 = 0 \tag{S34}$$

$$\omega_2 a_v (2\sigma - \sigma_1) - \frac{1}{4}\beta a_u^2 \cos\theta_1 - \frac{3}{4}\gamma a_u^2 a_v - \frac{3}{8}\gamma a_v^3 = 0 \tag{S35}$$

$$\omega_2 a_v \mu_2 + \frac{1}{4}\omega_2 a_v^3 \mu_{non} + \frac{1}{4}\beta a_u^2 \sin\theta_1 = 0 \tag{S36}$$

SUPPLEMENTARY NOTE III. IR-mediated damping analysis

0.1 Theoretical Basis of the RO Mode (a_u) Duffing Response

The standard Duffing response of the RO mode is primarily described by the frequency balance equation, Eq. S33. This equation balances the external drive detuning ($\omega_1 a_u \sigma$) against the internal frequency shifts. The crucial term is the cubic nonlinearity $-\frac{3}{8}\gamma a_u^3$, which acts as an amplitude-dependent stiffness (frequency shift).

To maintain steady-state, the total effective frequency detuning must be balanced:

$$\sigma_{\text{eff}} = \sigma - \frac{3\gamma a_u^2}{8\omega_1} - \ldots \text{(other coupling terms)} = 0$$

For a high-amplitude state ($a_u > 0$) and a hardening nonlinearity ($\gamma > 0$), the drive detuning σ must increase to compensate for the a_u^2 term. This relationship mathematically the resonance curve to the right (higher σ), creating the characteristic hardening backbone. The "jump frequency" observed experimentally is the point where this set of equations no longer possesses a stable, high-amplitude solution, which corresponds mathematically to a saddle-node bifurcation.

0.2 Theoretical Basis of the AP Mode (a_v) Parametric Response

The excitation mechanism of the AP mode is revealed by solving the AP mode equations, Eq. S35 and S36, for the amplitude a_v.

First, the AP energy balance equation Eq. S36 defines the phase lock θ_1 required to transfer energy and overcome damping:

$$\sin\theta_1 \approx -\frac{4\omega_2 a_v \mu_2}{\beta a_u^2} \tag{S37}$$

Second, the AP frequency balance equation S35 defines the phase lock for resonance. Ignoring γ terms for simplicity, we get:

$$\cos\theta_1 \approx \frac{4\omega_2 a_v(2\sigma - \sigma_1)}{\beta a_u^2} \tag{S38}$$

Using the trigonometric identity $\sin^2\theta_1 + \cos^2\theta_1 = 1$, we combine these two conditions to solve for a_v:

$$\left(-\frac{4\omega_2 a_v u_2}{\beta_2 a_u^2}\right)^2 + \left(\frac{4\omega_2 a_v(2\sigma - \sigma_1)}{\beta_2 a_u^2}\right)^2 \approx 1$$

This yields the approximate amplitude response for a_v:

$$a_v \approx \frac{\frac{1}{4}\beta a_u^2}{\sqrt{(\omega_2(2\sigma - \sigma_1))^2 + (\omega_2 u_2)^2}} \tag{S39}$$

This equation mathematically confirms the observed behavior: the AP mode amplitude a_v is parametrically pumped by a numerator proportional to a_u^2 (from the $\beta_2 a_u^2$ term) and is filtered by a Lorentzian-like denominator centered at the internal resonance condition $2\sigma - \sigma_1 = 0$ (i.e., $\delta \approx 0$).

0.3 Theoretical Mechanism of IR-mediated AP channel loss

The phenomenon of IR-mediated AP-channel loss, which is the root cause of the "clamped" response for $\delta > 0$, is a direct consequence of the energy feedback loop. This mechanism is mathematically proven by combining the energy balance equations for both modes, Eq. S34 and S36.

1. AP Mode Phase Lock: First, we analyze the AP energy balance, Eq. S36:

$$\omega_2 a_v u_2 + \frac{1}{4}\omega_2 a_v^3 \mu_{non} + \frac{1}{4}\beta a_v^2 \sin\theta_1 = 0$$

For the AP mode to exist ($a_v > 0$) and overcome its own intrinsic and nonlinear damping ($u_2 > 0, u_{non} > 0$), it must lock into a phase θ_1 such that $\sin\theta_1 < 0$ (assuming $\beta > 0$). This phase lock is a fundamental requirement for energy to be pumped from a_u to a_v.

2. RO Mode Energy Feedback: Next, we analyze the RO energy balance, Eq. S34, which can be rewritten to show the balance of power:

$$\frac{1}{2}F_1 \sin\theta_2 = \omega_1 a_u \mu_1 + \frac{1}{4}\omega_1 a_u^3 \mu_{non} - \frac{1}{2}\beta a_u a_v \sin\theta_1$$

This equation states that the power injected by the drive (F_1) must equal the power dissipated by intrinsic damping (μ_1, μ_{non}) plus the power fed back from the AP mode (the β term).

3. The AP channel loss Mechanism ($\sigma_1 > 0$): When $\sigma_1 > 0$, the system finds a stable solution where $a_v > 0$ after the main resonance. We can now substitute the phase condition from Step 1 ($\sin\theta_1 < 0$) into the RO energy balance equation from Step 2.

Assuming the coupling $\beta > 0$, the entire feedback term $-\frac{1}{4}\beta a_u a_v \sin\theta_1$ becomes a positive definite quantity. The RO energy balance becomes:

$$\underbrace{\frac{1}{2}F_1 \sin\theta_2}_{\text{Power In}} = \underbrace{\omega_1 a_u \mu_1 + \frac{1}{4}\omega_1 a_u^3 \mu_{non}}_{\text{Intrinsic Damping}} + \underbrace{\left| -\frac{1}{2}\beta_1 a_u a_v \sin\theta_1 \right|}_{\text{IR-mediated AP-channel loss}}$$

This equation is the mathematical proof of the phenomenon. The drive F_1 must now supply power to overcome both its own linear and nonlinear damping (μ_1,μ_{non}) and this new, powerful damping term created by the β feedback. This term, which is non-zero only when a_v is active, acts as a massive "energy cancellation" or "anti-drive" mechanism, suppressing the a_u amplitude. Therefore the total effective damping rate of RO mode under IR can be expressed as:

$$\Gamma_{\text{eff}} = 2\epsilon\omega_r \left(\mu_1 + \frac{1}{4}\mu_{\text{non}} a_u^2 - \frac{\beta a_v}{2\omega_1} \sin\theta_1 \right). \tag{S40}$$

Equation S40 defines the total effective damping rate, Γ_{eff}, of the RO mode under Internal Resonance (IR). This rate is composed of three distinct contributions as intrinsic linear damping, intrinsic nonlinear damping and IR-Enhanced damping. This third term is the mathematical manifestation of the IR-mediated AP-channel loss contribution. It acts as a variable dissipation channel that depends on the AP mode amplitude (a_v) and the relative phase difference (θ_1).

SUPPLEMENTARY NOTE IV. IR Frequency response and phase response

To provide a detailed physical mechanism for the experimentally observed phenomena, we analyze the numerical solutions to the slow-flow (static) equations (S33-36) under the three distinct internal detuning regimes ($\delta < 0$, $\delta \to 0$, and $\delta > 0$). The figures below plot the amplitudes a_u (RO mode) and a_v (AP mode), and the phases θ_1 and θ_2, as a function of the external drive detuning σ.

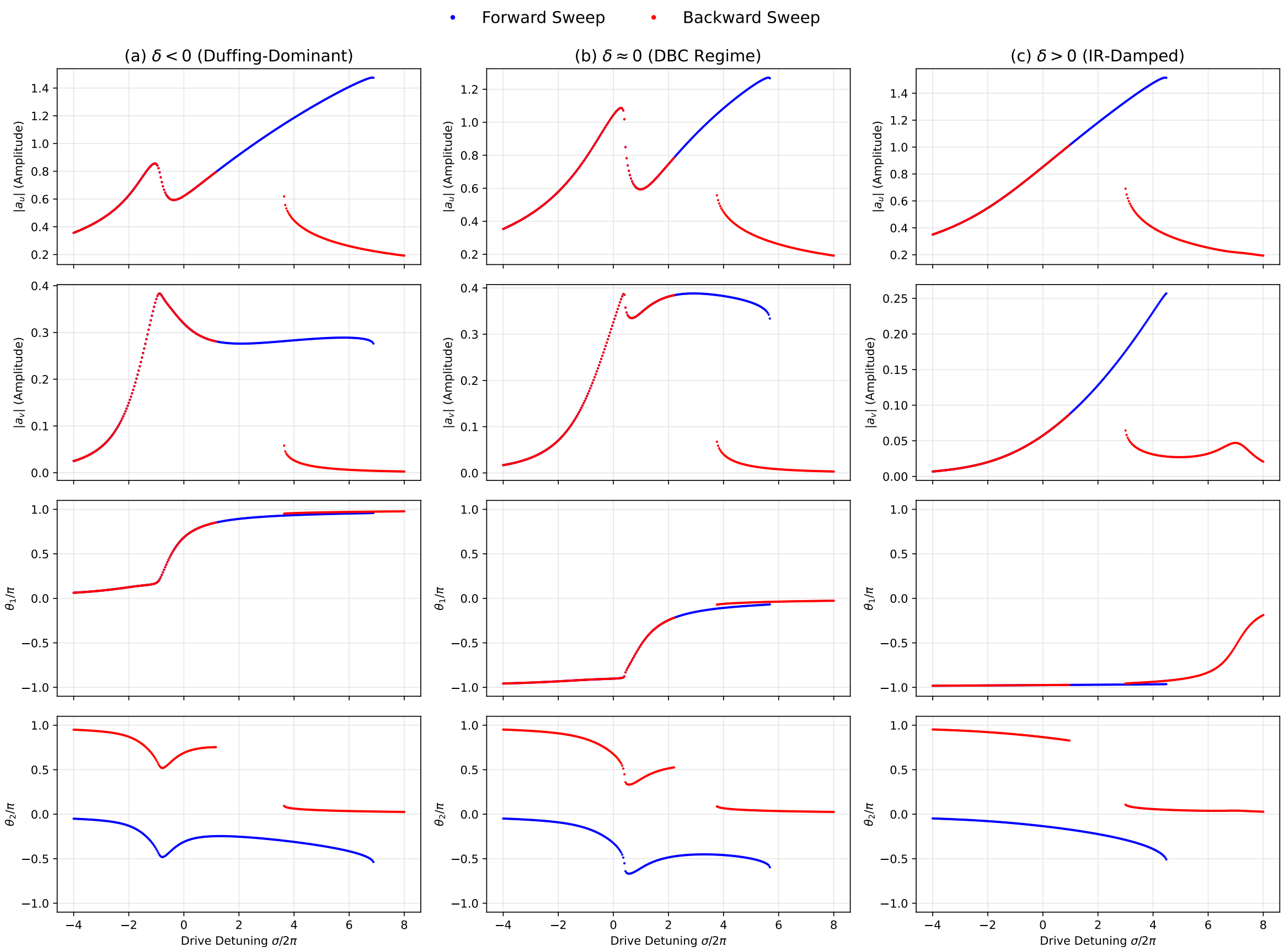

Figure S3: Numerical solutions of the averaged slow-flow equations (S33-36) showing the steady-state response evolution as a function of the internal detuning $\sigma_1 = (\omega_2 - 2\omega_1)/2\pi$. Each column represents a distinct dynamical regime: **(a)** $\sigma_1 < 0$ (Duffing-dominant), where the a_v resonance occurs at $\sigma < 0$, resulting in a weak coupling and a standard Duffing response for a_u. **(b)** $\sigma_1 \to 0$ (DBC Regime), where the a_u main resonance ($\sigma \approx 0$) and the a_v internal resonance ($2\sigma \approx \sigma_1$) collide, leading to unstable phase-locking (Hopf bifurcations) and the DBC. **(c)** $\sigma_1 > 0$ (IR-Damped), where the a_v resonance occurs at $\sigma > 0$. This establishes a stable phase lock (θ_1) that creates a powerful AP channel loss, "clamping" the a_u jump frequency at a lower value. Rows show the (Top) RO mode amplitude $|a_u|$, (Second) AP mode amplitude $|a_v|$, (Third) internal phase θ_1/π, and (Bottom) external phase θ_2/π. All plots show the forward sweep (blue dots) and backward sweep (red dots) versus the normalized drive detuning $\sigma/2\pi$. The hysteresis (separation between blue and red) visually represents the bistable region.

0.1 Case 1: $\sigma_1 < 0$ (Duffing-Dominant Regime)

As shown in Fig.S3a, this regime is "Duffing-dominant" because the two modes are largely decoupled.

Amplitude Response (a_u, a_v): The a_u amplitude exhibits a classic Duffing response. It follows a right-bending (hardening) backbone to a high amplitude before undergoing a sharp saddle-node bifurcation (jump) at $\sigma \approx 7$. This behavior is perfectly described by Eq. (S33), where the a_v coupling terms ($\gamma a_u a_v^2$, $\beta a_u a_v$) are negligible because a_v is small when $\sigma > 0$.

The a_v amplitude shows a weak, transient response. It peaks at $\sigma \approx -1$, which corresponds precisely to the AP mode's IR condition $2\sigma \approx \sigma_1$. This response is described by the derived Lorentzian expression (Eq.(S39) in our previous analysis), where a_v acts as a simple filter pumped by a_u^2. After its resonance, a_v remains near a constant amplitude as result of the duffing effect of a_u.

Phase Response (θ_1, θ_2): The external phase θ_2 follows the standard Duffing behavior: it slowly accumulates on the high-amplitude branch and then abruptly jumps at the bifurcation point. The internal phase θ_1 is only relevant where a_v is active (around $\sigma \approx -1$), showing a clear phase shift as it passes through its resonance.

0.2 Case 2: $\sigma_1 \to 0$ (Dissipative Bistability Collapse)

As shown in Fig.S3b, this is the most critical regime, where the a_u main resonance ($\sigma \approx 0$) and the a_v internal resonance ($2\sigma \approx \sigma_1 \approx 0$) collide.

Amplitude Response (a_u, a_v): This co-resonance creates the strongest pumping condition via the $\beta a_u a_v$ and βa_u^2 terms. The a_u energy is immediately and efficiently transferred to a_v. As seen in the figure, the a_v amplitude itself shows a strong hardening response (bending to the right), governed by its own cubic nonlinearities (γ terms in Eq. S35). This coupled hardening effect from both modes shifts the entire response to higher frequencies. As a result, the σ point where the DBC occurs is also "delayed" or pushed to a higher frequency (Fig.S3b, where collapse occurs near $\sigma/2\pi \approx 1$).

This intense interaction is fundamentally unstable. The drive (F_1) must overcome both intrinsic damping μ_1, μ_{non} and this new, powerful but unstable damping channel created by the $\beta a_u a_v$ feedback. This unstable energy transfer destroys the high-amplitude stable branch for a_u, leading to the DBC observed in the main text. This potent, instability-driven energy dissipation is the hallmark of the IR-mediated AP-channel loss in the $\sigma_1 \to 0$ regime.

Phase Response (θ_1, θ_2): The clearest evidence of this instability is in the phase plots. On the forward sweep (blue), both θ_1 and θ_2 fail to lock. They exhibit complex, unstable modulations and bifurcations (Hopf bifurcations) precisely where the stable high-amplitude branch would normally exist. The system cannot find a stable steady-state phase, leading to the collapse of the bistable region.

0.3 Case 3: $\sigma_1 > 0$ (IR Damped Regime)

As shown in Fig.S3c, this regime is defined by a stable, high-damping state caused by a unique phase-lock. The a_v resonance ($2\sigma \approx \sigma_1$) now occurs at a positive σ (e.g., $\sigma \approx 7$).

Amplitude Response (a_u, a_v): Both a_u and a_v (top two panels, blue curves) rise together along a coupled, hardening backbone. The jump frequency is "clamped" at a low value ($\sigma \approx 4.5$). The backward sweep (red curve) for a_v is particularly revealing: it shows the underlying Lorentzian resonance peak at $\sigma \approx 7$, which is "un-shrouded" after the coupled system has already jumped down.

Phase Response (θ_1, θ_2): The most critical feature is the internal phase θ_1 (third panel). On the high-amplitude branch ($\sigma < 4.5$), θ_1 is nearly flat, indicating a stable phase lock. This stable lock is the mathematical origin of the AP channel loss (a_v must lock to a phase where $\sin\theta_1 < 0.$).

0.4 Numerical and Experimental analysis

We systematically evaluated the IR frequency response of both the RO and AP modes across a range of detuning frequencies, δ. To understand the complex nonlinear dynamics described by the coupled equations of motion, Eqs. (S11) and (S12), we first performed numerical simulations using the Runge-Kutta method. The resulting 2D colormaps mapping amplitude against driving frequency and detuning, along with representative 1D frequency sweeps (cutlines), are shown in Fig. S4a. To validate these theoretical predictions, corresponding experimental measurements were conducted. The experimental colormaps and cutlines are presented in Fig. S4b. A comparison of Fig. S4a and Fig. S4b reveals a strong match between simulation and experimental results across varying δ. The 1D cutlines clearly illustrate three distinct dynamic regimes:

Duffing Dominant Regime ($\delta < 0$): Illustrated by the cutline at $\delta = -1.0$ Hz. In this region, the frequency mismatch is large enough that IR coupling is weak. The RO mode exhibits a classic hardening Duffing nonlinearity driven by the $r_{non}x_{RO}^2\dot{x}_{RO}$ and cubic stiffness terms.

DBC Regime ($\delta \to 0$): Illustrated by the cutline at $\delta = 0.5$ Hz. As detuning approaches zero, the strong 2:1 internal resonance dominates, leading to complex energy exchange and characteristic amplitude modulation features in the frequency response.

IR Damped Regime ($\delta > 0$): Illustrated by the cutline at $\delta = 7.0$ Hz. Here, the system is detuned again, but the presence of the AP mode manifests primarily as an increase in effective damping, suppressing the peak amplitude of the RO mode compared to the Duffing regime due

to the energy transfer mechanisms described in the equations of motion.

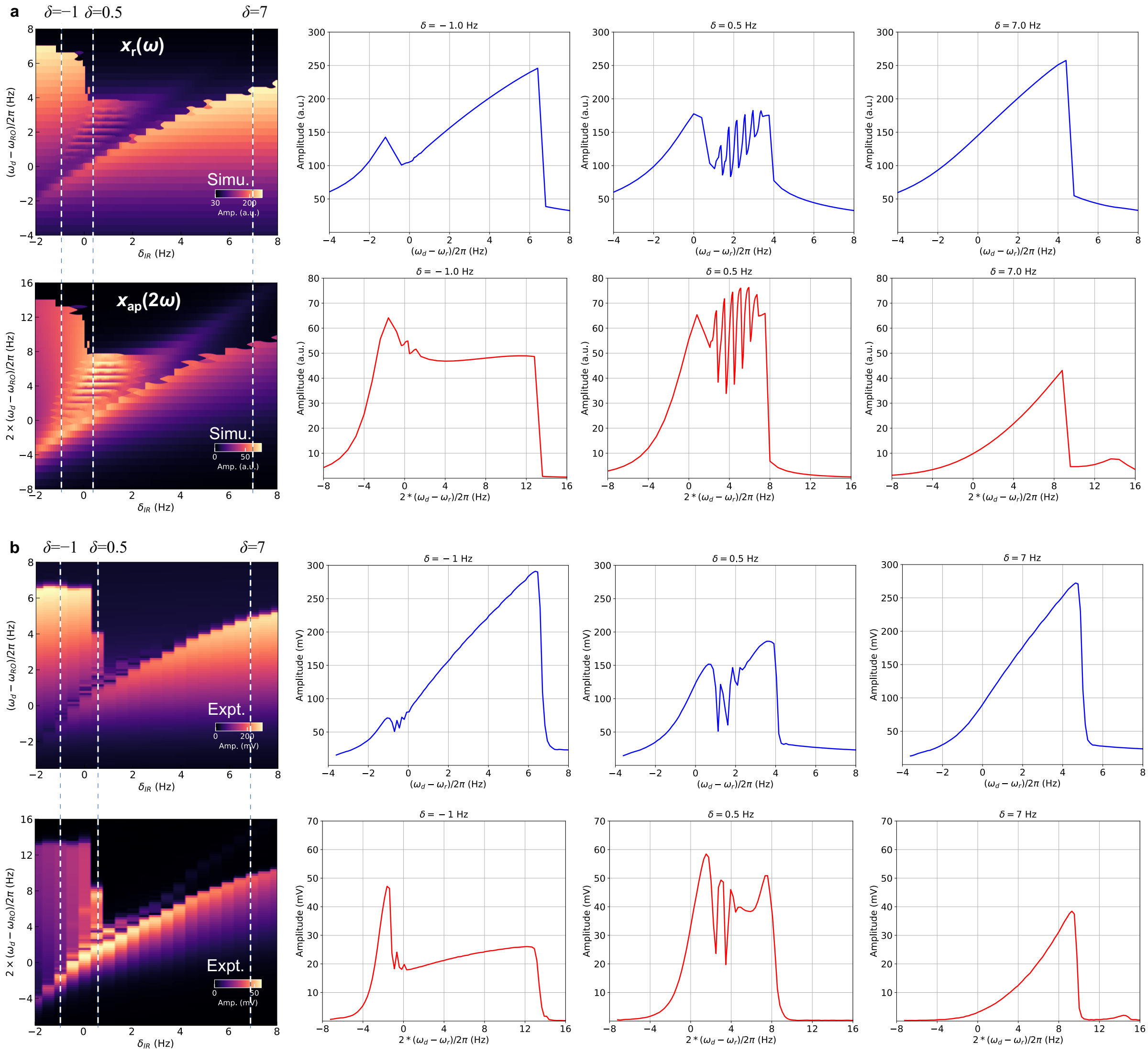

Figure S4: Simulation and experimental results of the IR response. (a) Simulation results visualizing the frequency response of the RO mode and AP mode under varying driving frequency and IR-resonance mismatch δ_{IR}. (b) Experimental results corresponding to the simulation parameters. The colormaps and 1D cutlines show the measured amplitude for the RO mode and AP mode at $\delta_{IR} = -1.0$ Hz, 0.5 Hz, and 7.0 Hz. The experimental data shows strong agreement with the numerical predictions across different dynamic regimes.

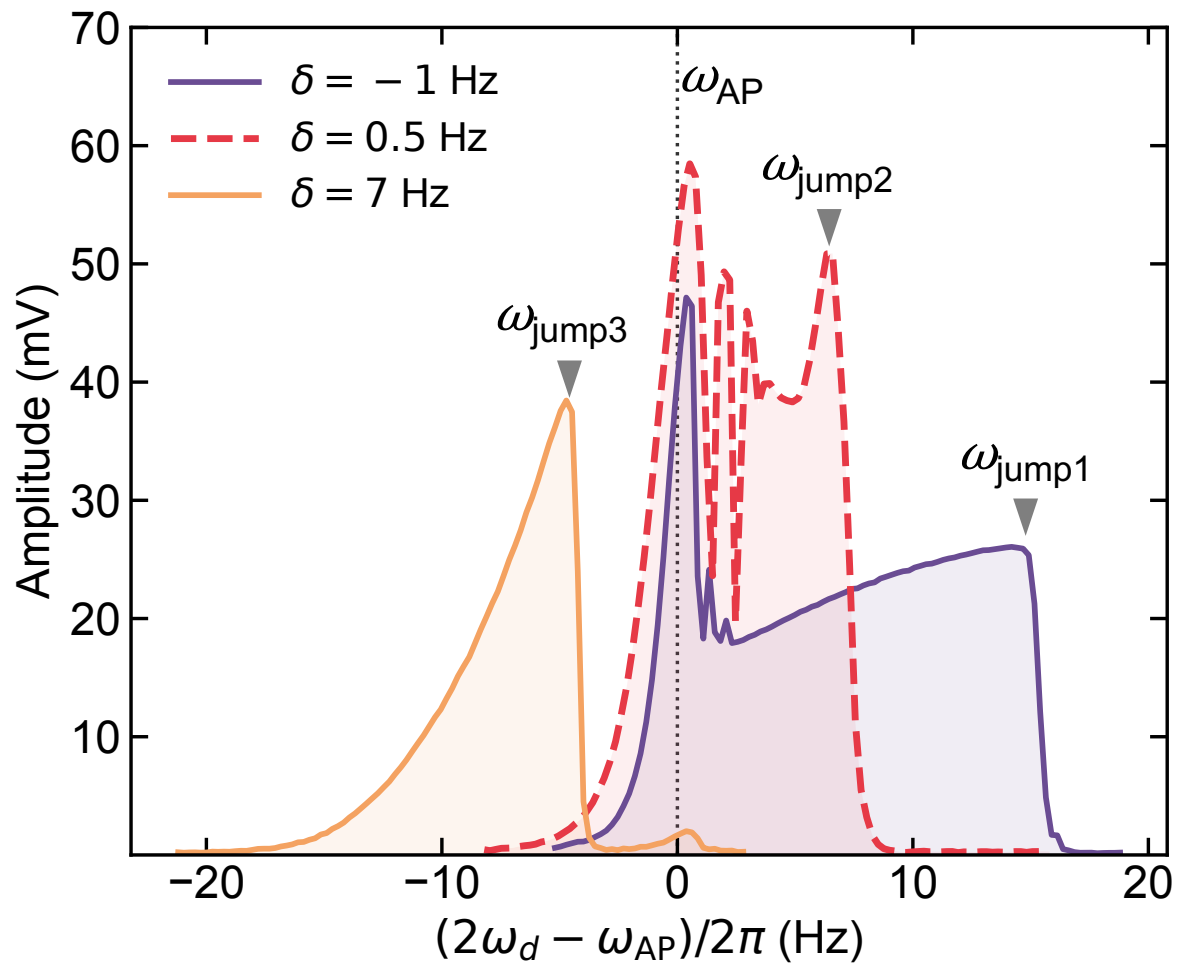

Figure S5: **AP-mode response replotted using the AP-referenced detuning axis.** The AP-mode response data (in Fig. 2b) are replotted as a function of the AP-referenced detuning, $(2\omega_d - \omega_{\mathrm{AP}})/2\pi$, where ω_d is the drive frequency applied to the RO mode and ω_{AP} is the electrostatically tuned AP-mode resonance frequency for each IR-mismatch condition. The three traces correspond to $\delta_{\mathrm{IR}} = -1, 0.5$, and 7 Hz, respectively. The vertical dotted line marks the AP resonance condition, $2\omega_d = \omega_{\mathrm{AP}}$. This representation shows that the AP response is organized by the second-harmonic internal-resonance condition. For negative IR mismatch, the AP response is excited near the AP resonance with Lorentz response but the RO Duffing branch remains largely intact resulting nearly flat response in AP output. Near zero IR mismatch, the AP response is strongly enhanced and develops modulation features associated with the DBC transition. For positive IR mismatch, the AP response is shifted and attenuated significantly, since the resonance happens after the RO jump.

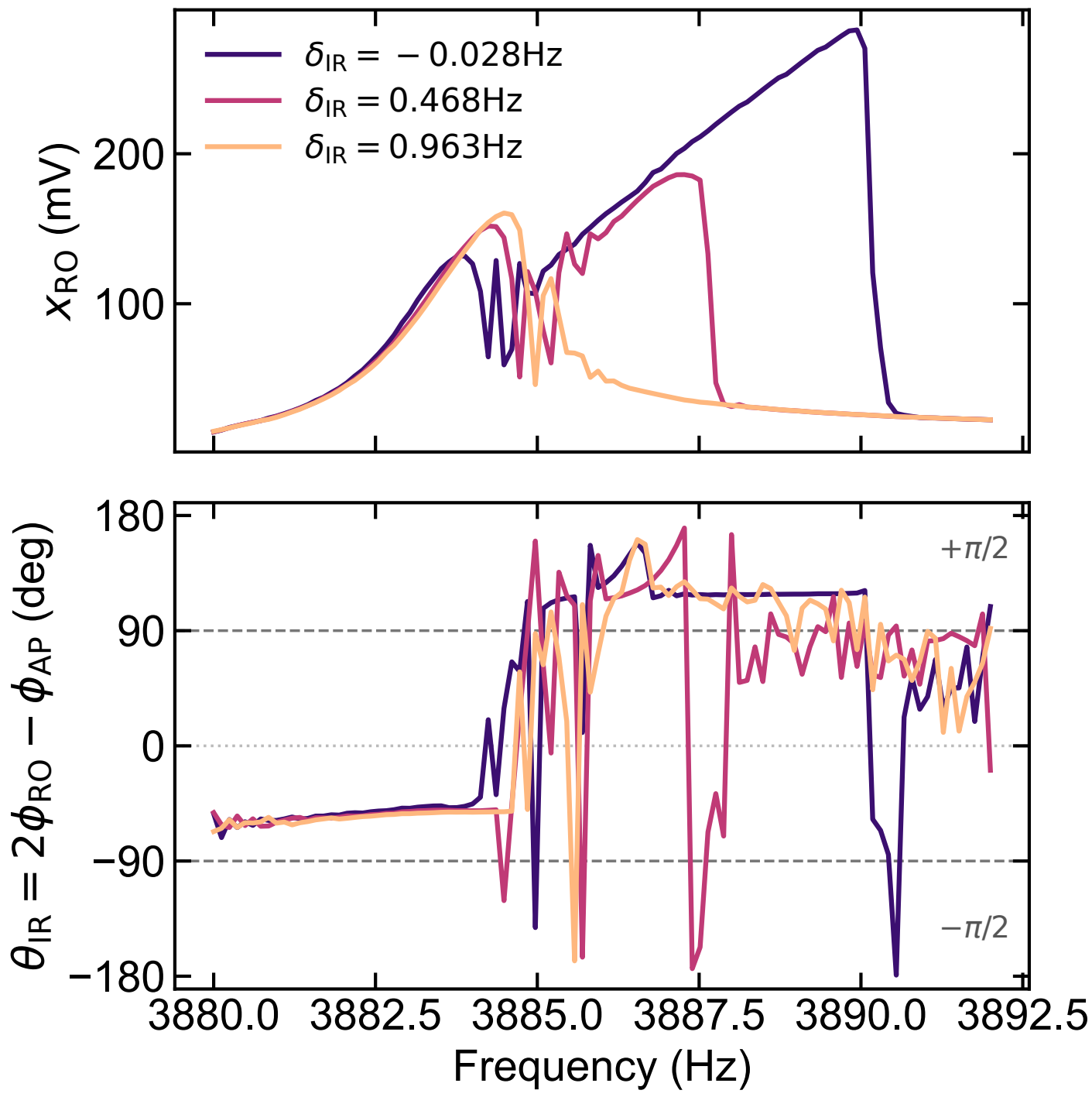

Figure S6: **Internal phase evolution near Hopf-type modulation region.** Measured RO amplitude and reconstructed internal phase mismatch $\theta_{\rm IR} = 2\phi_{\rm RO} - \phi_{\rm AP}$ for representative internal-resonance mismatch conditions. The phases are obtained from complex lock-in outputs, unwrapped along the frequency sweep, and wrapped to $[-\pi, \pi]$ for display. Strong phase modulation near the Hopf-type region reflects vigorous RO–AP energy exchange.
